# First Report of Potentially Pathogenic *Klebsiella pneumoniae* from Serotype K2 in Mollusk *Tegillarca granosa* and Genetic Diversity of *Klebsiella pneumoniae* in 14 Species of Edible Aquatic Animals

**DOI:** 10.3390/foods11244058

**Published:** 2022-12-15

**Authors:** Yingwei Xu, Ling Ni, Huiqiong Guan, Dailing Chen, Si Qin, Lanming Chen

**Affiliations:** 1 Key Laboratory of Quality and Safety Risk Assessment for Aquatic Products on Storage and Preservation (Shanghai), Ministry of Agriculture and Rural Affairs of the People’s Republic of China, College of Food Science and Technology, Shanghai Ocean University, Shanghai 201306, China; 2 East China Sea Fisheries Research Institute, Chinese Academy of Fishery Sciences, Shanghai 200090, China; 3 Lab of Food Function and Nutrigenomics, College of Food Science and Technology, Hunan Agricultural University, Changsha 410128, China

**Keywords:** *Klebsiella pneumoniae*, genetic diversity, virulence, antibiotic resistance, heavy metal tolerance, aquatic animal, food safety

## Abstract

*Klebsiella pneumoniae* can cause serious pneumonitis in humans. The bacterium is also the common causative agent of hospital-acquired multidrug-resistant (MDR) infections. Here we for the first time reported the genetic diversity of *K. pneumoniae* strains in 14 species of edible aquatic animals sampled in the summer of 2018 and 2019 in Shanghai, China. Virulence-related genes were present in the *K. pneumoniae* strains (*n* = 94), including the *entB* (98.9%), *mrkD* (85.1%), *fimH* (50.0%), and *ybtA* (14.9%) strains. Resistance to sulfamethoxazole-trimethoprim was the most prevalent (52.1%), followed by chloramphenicol (31.9%), and tetracycline (27.7%), among the strains, wherein 34.0% had MDR phenotypes. Meanwhile, most strains were tolerant to heavy metals Cu^2+^ (96.8%), Cr^3+^ (96.8%), Zn^2+^ (91.5%), Pb^2+^ (89.4%), and Hg^2+^ (81.9%). Remarkably, a higher abundance of the bacterium was found in bottom-dwelling aquatic animals, among which mollusk *Tegillarca granosa* contained *K. pneumoniae* 8-2-5-4 isolate from serotype K2 (ST-2026). Genome features of the potentially pathogenic isolate were characterized. The enterobacterial repetitive intergenic consensus polymerase chain reaction (ERIC-PCR)–based genome fingerprinting classified the 94 *K. pneumoniae* strains into 76 ERIC genotypes with 63 singletons, demonstrating considerable genetic diversity in the strains. The findings of this study fill the gap in the risk assessment of *K. pneumoniae* in edible aquatic animals.

## 1. Introduction

*Klebsiella pneumoniae* can cause life-threatening pneumonitis in humans [1,2]. The bacterium is also the common causative agent of nosocomial infection diseases, including septicemia, meningitis, osteomyelitis, thrombophlebitis, and urinary tract infections (UTIs) [3,4]. *K. pneumoniae* was initially isolated from the lungs of dead pneumonia patients in 1882 [5]. The bacterium is found growing in the environment, specifically in areas such as soil, water, and plant matter [6]. *K. pneumoniae* strains are classified into at least 79 serotypes, of which serotypes K1, K2, K5, K20, K54, and K57 are closely related to bacterial pathogenesis [7,8,9]. Studies have also indicated that K1 and K2 are high-risk clones with high virulence and multidrug resistance [10]. The rising incidence of high-virulent and multidrug-resistant (MDR) *K. pneumoniae* is one of the major clinical and public health issues worldwide [11].

Virulence determinants in *K. pneumoniae* include *aerobactin* (encoding the high-affinity iron chelator), *allS* (associated with the allantoin metabolism), *entB* (connected with the catecholate siderophore), *fimH* and *mrkD* (encoding fimbrial adhesion factors), *iroN* (related to the salmochelin biosynthesis), *magA* and *rmpA* (encoding regulators of mucoid phenotype), *traT* (encoding outer membrane proteins involved in surface exclusion), *wcaG* (related to outer-core lipopolysaccharide biosynthesis), and *ybtA* (involved in the acquisition of iron ions) [11,12,13,14,15]. For instance, regulators *magA* and *rmpA* are responsible for the hypermucoviscous phenotype of *K. pneumoniae*, while *fimH* and *mrkD* are adhesins that facilitate the bacterial adherence to the extracellular matrix to create biofilm [14]. The adhesion capability plays a crucial role in the colonization and invasion of pathogenic *K. pneumoniae* in the host.

Antibiotics are widely used to treat and prevent infectious diseases caused by pathogenic microorganisms. Nevertheless, the inappropriate utilization of antibiotics in medical treatment may lead to the emergence and spread of the MDR pathogen [8]. It was estimated that more than one-third of *K. pneumoniae* strains reported to the European Centre for Disease Prevention and Control (ECDC) were resistant to at least one antibiotic, including fluoroquinolones, third-generation cephalosporins, aminoglycosides, and carbapenems. The combined resistance to the former three antibiotics was the most common [16]. In China, the resistance rates of *K. pneumoniae* to imipenem (IPM) increased from 3% in 2005 to 25.3% in 2019 [17]. Moreover, the prevalence of carbapenems-resistant *K. pneumoniae* occurred in almost all provinces in China between 2010 and 2020 [17]. The MDR pathogenic *K. pneumoniae* poses serious threats to therapeutic options for human diseases [18,19].

On the other hand, heavy metals released from industries, agriculture, and human-made sources may result in the increased pollution in aquatic environments [20]. Because of long-term persistence and a nondegradable nature, heavy metals accumulate in humans, and this accumulation via food chain poses substantial risks to human health [21]. Heavy metal residues have been reported in many aquatic products, particularly in developing nations. For instance, it was recently reported that 100%, 100%, 77.4%, and 34.0% of aquatic product samples (*n* = 108), which were collected during the summers of 2018 and 2019 in Shanghai, China, tested positive for the cadmium (Cd), copper (Cu), mercury (Hg), and lead (Pb) residues, respectively. Although none of these residues surpassed individual maximum residue limits (MRLs) [22], e.g., the MRLs of Cd in fish, mollusks, and crustaceans are 0.1 mg/kg, 1.0 mg/kg, and 0.5 mg/kg, respectively, critical concentrations of heavy metal accumulation could promote the spread of antibiotic resistance through coselection mechanisms [23].

A number of *K. pneumoniae* strains have been isolated from clinical samples and characterized [24,25]. However, to the best of our knowledge, the current literature in the prevalence and genetic diversity of *K. pneumoniae* in aquatic animals is little [26]. Recently, Håkonsholm et al. [27] isolated *K. pneumoniae* strains (*n* = 78) from *Mytilus edulis*, *Crassostrea gigas*, *Pecten maximus*, and *Politapes rhomboides*, which were collected along the Norwegian coast from September 2019 to March 2020. They found that 94.9% of these strains were resistant to ampicillin (AMP); 3.8% resistant to amoxicillin-clavulanic acid (AMC), tetracycline (TET), and piperacillin-tazobactam (TZP); 2.6% resistant to chloramphenicol (CHL), nitrofurantoin (NIT), and trimethoprim-sulfamethoxazole (SXT); and 1.3% resistant to aztreonam (ATM), ciprofloxacin (CIP), cefotaxime (CTX), and cefuroxime (CXM) [27]. Bacterial resistance genes derived from aquatic environments could be horizontally transferred to other animals and humans through the food chain, air, water, or manured and sludge-fertilized soils [28]. For example, the *TolC* gene, which encodes a major mediator of antibiotic resistance in *K. pneumoniae* for the outer membrane efflux channel, is implicated in the bacterium’s drug resistance to a variety of classes [29]. The *TEM-116* and *SHV-38* genes encoding extended-spectrum lactamases (ESBLs) can hydrolyze penicillins, cephalosporins, and monobactams [30].

The new finding of *K. pneumoniae* present in commonly edible aquatic animals poses tremendous danger to food safety and human health. Person-to-person or person-to-item interaction during food preparation may transmit *K. pneumoniae* [31]. Temperatures over 35 °C have been shown to reduce *K. pneumoniae* growth and metabolic activity. At 60 °C, there is a significant drop in growth; however, *K. pneumoniae* still exhibits some metabolic activity (i.e., not completely inactivated) [32]. Food handlers should thus be aware of their involvement in the spread (or control) of the pathogenic bacterium.

China is the world’s largest producer, exporter, and consumer of aquatic products and has contributed approximately 60% (64,636,700 tons) to the global output in 2021 (National Bureau of Statistics, http://www.stats.gov.cn/ (accessed on 1 August 2022)). Continuous monitoring and identification of risk factors in the aquatic products are crucial for food safety and human health. Thus, the major objectives of this study were to survey the prevalence, virulence, and resistance of *K. pneumoniae* in commonly the consumed 41 species of aquatic animals collected in Shanghai, China. Of these, 39 species have not ever been detected having *K. pneumoniae*. Genetic diversity and coresistance between antibiotics and heavy metals of 94 *K. pneumoniae* strains recovered in the survey were also investigated. The findings of this study will fill gaps in the risk assessment of *K. pneumoniae* in edible aquatic animals.

## 2. Materials and Methods

### 2.1. Sample Collection

The 41 species of aquatic animals included mollusks (*n* = 21), fish (*n* = 17), and crustaceans (*n* = 3) (Table S1). Samples (*n* = 108) were collected in sterile plastic bags (Nanjing Maojie Microbial Technology Co., Ltd., Nanjing, China) from the Shanghai Jiangyang Aquatic Market and Shanghai Luchao Port Aquatic Market in July, August, and September of 2018 and 2019 in Shanghai, China [22]. The samples were stored in iceboxes (700 × 440 × 390 mm) and immediately transported to the laboratory at Shanghai Ocean University (Shanghai, China) for analysis within 24 h at 4 °C [22].

### 2.2. Isolation and Identification of K. pneumoniae

*K. pneumoniae* was isolated and identified in accordance with the National Standards of the People’s Republic of China (SN/T 1962–2007). The samples were pretreated as described in our recent research [33]. Briefly, an aliquot (25 g) of each sample was mixed with 225 mL of 1× phosphate-buffered saline (PBS, pH 7.4–7.6, Shanghai Sangon Biological Engineering Technology and Services Co., Ltd., Shanghai, China), homogenized, serial diluted, and spread onto the MacConkey Inositol Adonitol Carbenicillin (MIAC, Beijing Land Bridge Technology Co., Ltd., Beijing, China) agar plates [34]. The red colonies grown on the MIAC agar plates at 37 °C were randomly picked out for further identification. *K. pneumoniae* ATCC13883 was used as the control strain.

The matrix-assisted laser desorption/ionization time-of-flight mass spectrometry (MALDI-TOF/MS) platform (Microflex LT/SH, MALDI Biotyper, Bruker, Germany) was used to identify presumptive *K. pneumoniae* colonies; the platform was run under the same conditions and parameters in our recent studies [34,35]. Mass spectra were collected and analyzed by using the Microflex LT Mass Spectrometer (Bruker 422 Daltonics, Bremen, Germany) [34,35].

Biochemical tests, including capsular staining, Gram’s staining, and dynamic tests (Qingdao Haibo Bio Co., Ltd., Qingdao, China), were also used to identify *K. pneumoniae* strains, in accordance with the manufacturer’s instructions and the Chinese Government Standard (SN/T 1962–2007).

*K. pneumoniae* strains were also confirmed using the polymerase chain reaction (PCR) assay to amplify the 16S rRNA gene with the universal bacterial primers (Table S2) and DNA sequencing analysis [33,34].

### 2.3. Identification of Virulence Genes

Virulence genes (*aerobactin*, *alls*, *entB*, *fimH*, *iroN*, *magA*, *mrkD*, *rmpA*, *traT*, *wcaG*, and *ybtA*) were detected using the PCR assay [15,36,37,38,39,40,41]. All oligonucleotide primers (Table S2) used in this study were synthesized by the Sangon (Shanghai, China). The genomic DNA of strains was prepared using the TaKaRa MiniBEST Bacterial Genomic DNA Extraction Kit version 3.0 (TaKaRa Biomedical Technology Co., Ltd., Beijing, China). The 20 μL PCR reaction mixture contained 8 μL of DNase/RNase-free deionized water (Tiangen Biotech Co., Ltd., Beijing, China), 10 μL of 2× Taq Master Mix (Novoprotein Technology Co., Ltd., Shanghai, China), 0.5 μL of each primer (5 μM), and 1 μL of DNA template. PCR amplification was performed as described in our previous research [33,34,42], but with different annealing temperatures and elongation times, based on the melting temperatures of the primer pairs and the predicted sizes of the PCR products (Table S2). The obtained PCR products were analyzed as previously described [33,34,42]. DNA sequencing was carried out by Suzhou Jinweizhi Biotechnology Co., Ltd., Suzhou, China. The basic local alignment search tool (BLAST) of National Center for Biotechnology Information (NCBI) was used for sequence analysis (https://www.ncbi.nlm.nih.gov/ (accessed on 19 August 2021)). The virulence gene sequences of *K. pneumoniae* in the NCBI sequence databases were compared in the BLAST analysis. The standard strain *K. pneumoniae* ATCC13883 (Guangdong Culture Collection Center, Guangzhou, China) was used as a positive control strain in this study.

### 2.4. Enterobacterial Repetitive Intergenic Consensus-PCR (ERIC-PCR) Assay

The ERIC-PCR was used for genotyping of *K. pneumoniae* strains as described in previous studies (Table S2). The 20 μL reaction mixture contained 6 μL of DNase/RNase-free deionized water (Tiangen Biotech Co., Ltd., Beijing, China), 10 μL of 2 × Taq Master Mix (Novoprotein Technology, Shanghai, China), 1 μL of each primer (5 μM), and 2 μL of a DNA template. ERIC-PCR reactions were performed using the primers ERIC1R and ERIC2 (Table S2) at 95 °C for 30 s, 52 °C for 1 min, and at 65 °C for 8 min for 32 reaction cycles [34]. Amplified DNA fragments were observed, recorded, and analyzed; the unweighted pair group with arithmetic averages (UPGMA) method was used for cluster analysis; and the Simpson index was calculated to assess the diversity of *K. pneumoniae* strains [33,34,42,43].

### 2.5. Antibiotic Susceptibility and Heavy Metal–Tolerance Assays

The antibiotic susceptibility of *K. pneumoniae* strains was examined according to the method issued by the Clinical and Laboratory Standards Institute (CLSI, M100-S28, 2018). Antimicrobial agents included CHL (30 μg), CIP (5 μg), gentamicin (GEN, 10 μg), IPM (10 μg), kanamycin (KAN, 30 μg), meropenem (MEM, 10 μg), norfloxacin (NOR, 10 μg), SXT (23.75 μg sulphamethoxazole-1.25 μg trimethoprim), and TET (30 μg) (Oxoid, Basingstoke, UK). The MDR phenotype was defined when a strain was resistant to two or more antimicrobial agents. The minimal inhibitory concentrations (MICs) of eight heavy metals were determined using broth dilution testing (microdilution) [33,34,42]. CdCl_2_, CrCl_3_, CuCl_2_, HgCl_2_, MnCl_2_, NiCl_2_, PbCl_2_, and ZnCl_2_ were purchased from the Sinopharm (Shanghai, China). *K. pneumoniae* ATCC13883 and *E. coli* K12 (Institute of Industrial Microbiology, Shanghai, China) were used as quality-control strains.

### 2.6. Serotyping of K. pneumoniae Strains

The serotypes (K1, K2, K5, K20, K54, and K57) associated with the pathogenesis of *K. pneumoniae* were detected using the PCR assay with the primers in Table S2 [9,44]. The PCR reactions were performed, and PCR products were analyzed as described previously [33,34,42].

### 2.7. Genome Sequencing, Assembling and Annotation

The genome of *K. pneumoniae* 8-2-5-4 strain was sequenced by Shanghai Majorbio Bio-Pharm Technology Co., Ltd. (Shanghai, China) using the Illumina Hiseq 10 platform (Illumina, San Diego, CA, USA). The sequencing reads were approximately 150 bp in size (on average). Low-quality sequencing reads were filtered, and high-quality reads were assembled using the SOAPdenovo (version 2.04) software under the parameters described in our previous study [45]. The coding sequences (CDSs), rRNA, and tRNA genes were predicted using Glimmer software (version 3.02) [46], the Barrnap (https://github.com/tseemann/barrnap (accessed on 2 April 2022)) and tRNAscan-SE (version 2.0) software [47], respectively. The *K. pneumoniae* subsp. *pneumoniae* HS11286 genome (GenBank accession no. CP003200.1) was used as the reference genome.

Functional assignments were inferred using the standalone BLAST software [48]. The virulence factor database (http://www.mgc.ac.cn/VFs (accessed on 2 April 2022)), the comprehensive antibiotic resistance database (CARD, http://arpcard.Mcmaster.ca, Version 1.1.3) [49], and the BacMet database (http://bacmet.biomedicine.gu.se/ (accessed on 21 September 2022)) were utilized to identify virulence, antibiotic and heavy metal resistance–related genes, respectively. Mobile genetic elements (MGEs) were described and identified according to the methods described in our recent research [48]. The software was run with the default settings.

### 2.8. Multilocus Sequence Typing (MLST) Analysis

For seven conserved core genes, namely *gapA*, *infB*, *mdh*, *pgi*, *phoE*, *rpoB*, and *tonB*, in *K. pneumoniae*, the MLST analysis of the genome sequence of *K. pneumoniae* 8-2-5-4 strain was performed using the MLST database (https://cge.food.dtu.dk/services/MLST/ (accessed on 23 November 2022)).

### 2.9. Statistical Analysis

The SPSS version 17.0 software (SPSS Inc., Chicago, IL, USA) was used for statistical analysis. The multiple antibiotic resistance index (MARI) values were calculated according to the formula described previously [50]. In this study, all tests were performed in triplicate.

## 3. Results

### 3.1. K. pneumoniae in the 41 Species of Aquatic Animals

*K. pneumoniae* in the 41 species of aquatic animals was isolated, of which 39 species have not ever been detected for the bacterium, except *Mytilus edulis* and *Ostrea gigas thunberg* (Table S1). Approximately 567 red colonies were picked from the MIAC agar plates, and 16.6% (*n* = 94) of the colonies were identified as *K. pneumoniae* by the MALDI-TOF/MS analysis. The 94 *K. pneumoniae* strains also tested positive for the capsular staining and negative for the Gram’s staining and dynamic testing. The sequencing of the bacterial 16S rRNA gene validated the results as well (Table S3).

The 63.8% (*n* = 60), 22.3% (*n* = 21), and 13.8% (*n* = 13) of the 94 *K. pneumoniae* strains were isolated from the mollusks, fish, and crustaceans, respectively. Approximately 62.8% of the *K. pneumoniae* strains originated from eight species of saltwater animals and 37.2% from six species of freshwater animals. Remarkably, the majority of the *K. pneumoniae* strains (86.2%, 81/94) were isolated from the benthic animals, including nine species of mollusks (*n* = 60) and two species of crustaceans (*n* = 21). Of these, the top three benthic animals containing more *K. pneumoniae* strains were *Cipangopaludina cahayensis* (*n* = 30), *Procambarus clarkii* (*n* = 15) and *Mactra antiquata* (*n* = 13) (Table S1).

The 94 *K. pneumoniae* strains were present in 14 species of aquatic animals, including nine species of mollusks (*M. antiquata*, *Babylonia areolata*, *C. cahayensis*, *Neptunea cumingi* Crosse, *Tegillarca granosa*, *Haliotis rubra*, *Mactra veneriformis*, *Solen strictus*, and *Anodonta woodiana*); two species of crustaceans (*Eriocheir sinensis* and *P. clarkii*); and three species of fish (Blotchy rock cod, *Misgurnus anguillicaudatus*, and *Carassius auratus*) (Table S1). To the best of our knowledge, no literature is available on *K. pneumoniae* isolated from these species of aquatic animals, except *P. clarkii*.

The 94 *K. pneumoniae* strains were isolated from the aquatic animals originating from different regions in China, including Shanghai City and the Fujian, Zhejiang, Jiangsu, Shandong, and Hunan provinces. The highest percentage of *K. pneumoniae* were found in the samples from Shandong Province (31.9%, 30/94), followed by those from Shanghai City (22.3%, 21/94), Zhejiang Province (14.9%, 14/94), and Hunan Province (13.8%, 13/94). Only a few strains were recovered from the samples originating from Fujian Province (8.5%, 8/94) and Jiangsu Province (8.5%, 8/94). Additionally, approximately 56.4% (53/94) of the *K. pneumoniae* strains were collected from the Luchao Port Aquatic Market and 43.6% (41/94) from the Jiangyang Aquatic Market in Shanghai, China (Table S4).

### 3.2. Virulence-Related Genes in the K. pneumoniae Strains by PCR Assay

All the 94 *K. pneumoniae* strains tested negative for the virulence-related genes *aerobactin*, *allS*, *iroN*, *rmpA*, *traT*, and *wcaG* using the PCR assay. However, higher incidences of the *entB* (98.9%) and *mrkD* (85.1%) genes were observed, followed by the *fimH* (50.0%) and *ybtA* (14.9%) genes among the *K. pneumoniae* strains. The amplified products of the *entB*, *mrkD*, *ybtA*, and *fimH* genes were confirmed by DNA sequencing (Table S3).

The *K. pneumoniae* strains in the 14 species of aquatic animals showed different virulence gene profiles (Table 1). Higher percentages of the *entB* (100.0%), *mrkD* (78.3%), and *fimH* (56.7%) genes were detected in the *K. pneumoniae* strains in the mollusks. The former two genes were also found in most strains from the crustaceans (100.0% and 100.0%) and fish (92.3% and 92.3%) samples. The *ybtA* gene was found only in the strains from the crustaceans (38.1%) and mollusks (10.0%). Notably, three *K. pneumoniae* strains (7-17-4, 7-17-6, and 7-17-8) isolated from the mollusk *C. cahayensis* carried the four virulence genes (*entB*, *mrkD*, *ybtA*, and *fimH*).

The 94 *K. pneumoniae* strains had nine virulence-related gene profiles (Table 2). All the strains were mostly detected by the presence of the *entB* gene alone or in combination with other genes (*mrkD*, *ybtA*, and *fimH*). For example, the three strains (7-17-4, 7-17-6, and 7-17-8) had the *entB^+^*/*fimH^+^*/*mrkD^+^*/*ybtA^+^* gene profile. Approximately 38 and 9 *K. pneumoniae* strains harbored the *entB^+^/fimH^+^/mrkD^+^* and *entB^+^/mrkD^+^/ybtA^+^* gene profiles, respectively (Table 2).

These results indicated a variable risk of potentially pathogenic *K. pneumoniae* strains in the 14 species of edible aquatic animals.

### 3.3. Antimicrobial-Resistance Profiles of the K. pneumoniae Strains

The antimicrobial susceptibility of the 94 *K. pneumoniae* strains was examined (Table 3). All the *K. pneumoniae* strains were sensitive to MEM (100%); the majority of the strains were sensitive to IPM (98.9%), GEN (84.0%), NOR (81.9%), CIP (68.1%), and KAN (63.8%); and 30.9% of the strains were susceptible to all the antibiotics evaluated. Conversely, the SXT resistance rate was the highest (52.1%) among the *K. pneumoniae* strains, followed by CHL (31.9%) and TET (27.7%). Approximately 18.1% and 13.8% of the *K. pneumoniae* strains also showed intermediate susceptibility to CIP and KAN.

*K. pneumoniae* strains in the mollusks, fish, and crustaceans had different antibiotic resistance profiles (Table 4). Approximately 69.2% of the *K. pneumoniae* strains (*n* = 13) in the fish were resistant to SXT, followed by 56.7% (*n* = 60) from the mollusks, and 28.6% (*n* = 21) from the crustaceans. Higher resistance rates were also observed in TET (69.2%), CHL (61.5%), CIP (61.5%), KAN (61.5%), and NOR (46.2%) among the strains from the fish than those from the crustaceans (9.5%, 19%, 4.8%, 4.8%, and 4.8%) and mollusks (25.0%, 30.0%, 6.7%, 20.0%, and 1.7%). Additionally, the GEN resistance (10.0%) was found only in the strains from the mollusks.

On the basis of the limited numbers of *K. pneumoniae* strains obtained in this study, distinct antibiotic resistance profiles were also found among the *K. pneumoniae* strains in the 14 species of aquatic animals (Table 4). For example, all the *K. pneumoniae* strains (*n* = 6) in *M. anguillicaudatus* were resistant to CHL, CIP, KAN, NOR, TET, and SXT. The strains from *S. strictus* (*n* = 3), *M. veneriformis* (*n* = 2), and *B. areolate* (*n* = 1) were all resistant to SXT. Conversely, the resistance to GEN was present only in a few strains from *C. cahayensis* (2/30), *H. rubra* (1/5), *M. antiquata* (2/13), and *M. veneriformis* (1/2).

### 3.4. MDR Phenotypes of the K. pneumoniae Strains

Approximately 34.0% (32/94) of the *K. pneumoniae* strains exhibited MDR phenotypes. The highest percentage of MDR strains (69.2%, 9/13) was found in the fish, followed by the mollusks (31.7%, 19/60) and the crustaceans (19.0%, 4/21). The MDR phenotypes were predominant among the *K. pneumoniae* strains in *M. anguillicaudatus* (100%, 6/6) and *M. veneriformis* (100%, 2/2), followed by *M. antiquata* (76.9%, 10/13), *E. sinensis* (66.7%, 4/6), *S. strictus* (66.7%, 2/3), *B. rock cod* (50.0%, 3/6), and *H. rubra* (40.0%, 2/5). In contrast, only a few *K. pneumoniae* strains (10.0%, 3/30) in *C. cahayensis* showed MDR phenotypes, and the other six species of aquatic animals were devoid of MDR *K. pneumoniae* strains.

The MARI values of the 94 *K. pneumoniae* strains ranged from 0.00 to 0.67. The maximum MARI value was observed from the *K. pneumoniae* strains in *M. anguillicaudatus* (0.67), followed by *M. veneriformis* (0.39), *M. antiquata* (0.34), and *S. strictus* (0.34), whereas the strains in *C. cathayensis*, *T*. *granosa*, and *P. clarkii* had the smaller MARI values of 0.08, 0.07, and 0.01, respectively. Notably, *K. pneumoniae* 8-1-13-1 isolate from *E. sinensis; K. pneumoniae* 8-1-5-1, 8-1-5-2, 8-1-5-3, 8-1-5-4, 8-1-5-5, and 8-1-5-6 strains from *M. anguillicaudatus;* and *K. pneumoniae* 8-2-1-9 isolate from *M. veneriformis* had the highest MARI value, of 0.67, and were resistant to six antimicrobial agents.

### 3.5. Heavy Metal–Tolerance Profiles of the K. pneumoniae Strains

Tolerance of the 94 *K. pneumoniae* strains to eight heavy metals was also examined (Table S5). The maximum MICs of the *K. pneumoniae* strains were 3200 μg/mL for Cu^2+^, Cr^3+^, Mn^2+^, Pb^2+^, and Zn^2+^; 800 μg/mL for Ni^2+^, and Cd^2+^; and 50 μg/mL for Hg^2+^, when compared with the quality-control strain, *E. coli* K12. The Cu^2+^ and Cr^3+^ tolerances were predominant among the *K. pneumoniae* strains (96.8%, and 96.8%), followed by Zn^2+^ (91.5%), Pb^2+^ (89.4%), Hg^2+^ (81.9%), Cd^2+^ (33.0%), and Mn^2+^ (14.9%). Conversely, all the *K. pneumoniae* strains were sensitive to Ni^2+^.

**Figure 1:**
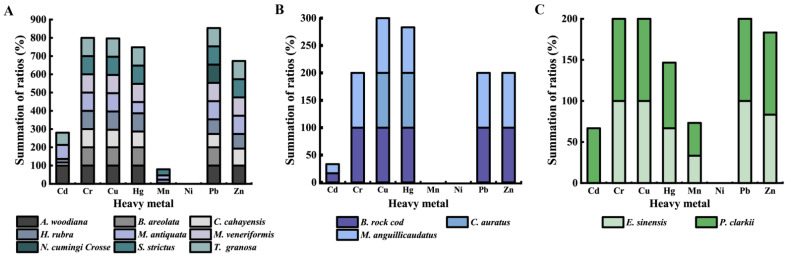
The heavy metal–tolerance profiles of the 94 *K. pneumoniae* strains in the 14 species of aquatic animals: mollusks (**A**), fish (**B**), and crustaceans (**C**).

*K. pneumoniae* strains in the mollusks, fish, and crustaceans had distinct heavy metal–tolerance profiles (Figure 1). Notably, most *K. pneumoniae* strains from all the aquatic products tolerated Cr^3+^ (100% to 92.3%), Cu^2+^ (100% to 95.0%), Hg^2+^ (92.3% to 76.2%), Pb^2+^ (100% to 85.0%), and Zn^2+^ (95.2% to 90.0%). Moreover, the highest percentages of Cd^2+^- and Mn^2^+-tolerant strains (47.6% and 38.1%) were observed in the crustaceans, followed by the mollusks (31.7% and 10.0%) and the fish (15.4% and 0.0%).

The *K. pneumoniae* strains in the 14 species of aquatic animals also had different heavy metal–tolerance profiles (Figure 1). For instance, the *K. pneumoniae* strains in *M. antiquata*, *C. cahayensis*, *P. clarkii*, and *H. rubra* showed tolerance to seven heavy metals, followed by *A. woodiana*, *B. rock cod*, *E. sinensis*, *M. anguillicaudatus*, *S. strictus*, and *T. granosa* (six heavy metals); *M. veneriformis* (five heavy metals); and *B. areolata* (four heavy metals), *C. auratus* (two heavy metals), and *N. cumingi Crosse* (one heavy metal).

### 3.6. Genetic Diversity of the K. pneumoniae Strains

**Figure 2:**
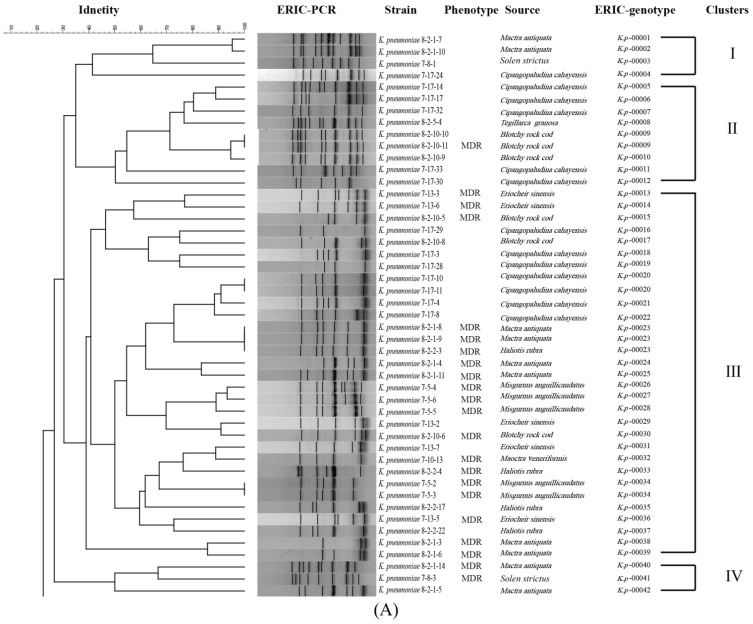

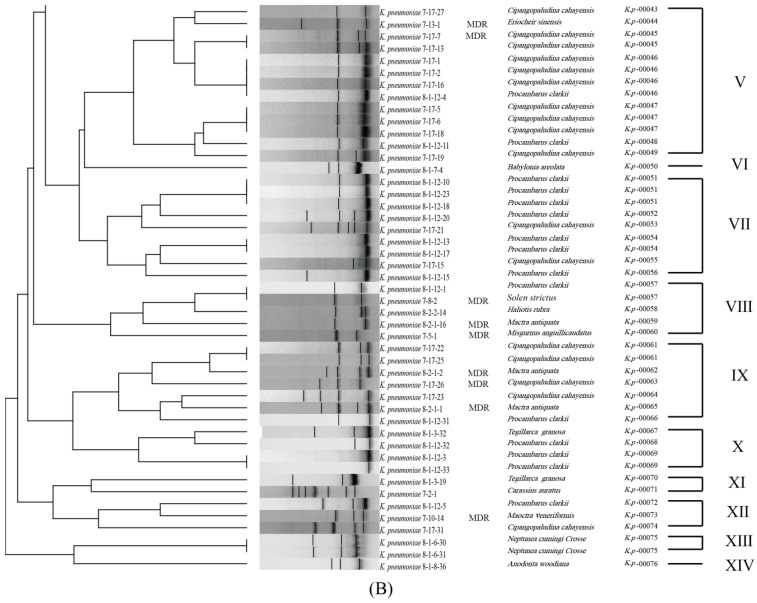
The ERIC-PCR-based genotyping of the 94 *K. pneumoniae* strains (**A**,**B**).

The ERIC-PCR was used for the genotyping of the 94 *K. pneumoniae* strains (Figure 2). On the basis of the fingerprinting profiles, the 94 *K. pneumoniae* strains were classified into 76 distinct ERIC genotypes, wherein 82.9% (63/76) were classified as singletons. Approximately 28.5% (18/63), 9.52% (6/63), 17.5% (11/63), and 9.52% (6/63) of these singletons were derived from *C. cahayensis*, *E. sinensis, M. antiquata*, and *P. clarkii*, respectively. The 76 ERIC genotypes belonged to 14 distinct clusters (Clusters I to XIV). Approximately 33.0% (31/94) of the *K. pneumoniae* strains fell into the largest, Cluster III, followed by 13.8% (13/94), 9.57% (9/94), and 9.57% (9/94) into Cluster V, Cluster II, and Cluster VII, respectively. The remaining (34.1%) were grouped into the other 10 clusters (7.45% to 1.06%). The majority of *K. pneumoniae* strains had similarity coefficients from 30.0% to 90.0% a Simpson’s diversity index of 0.8499. These results demonstrated genetic variation among the 94 *K. pneumoniae* strains in the 14 species of aquatic animals.

### 3.7. Heavy Metal Tolerance and MDR of the K. pneumoniae Strains

**Figure 3:**
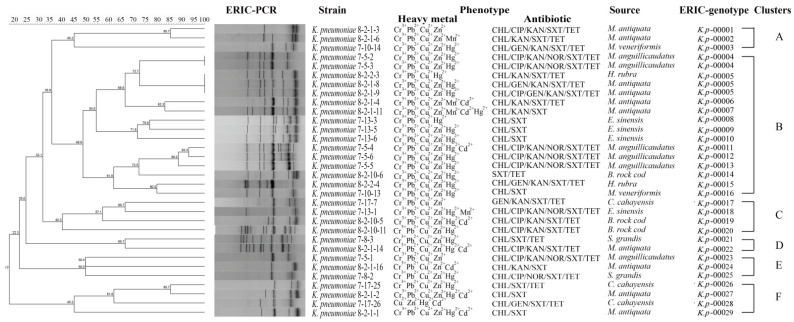
The ERIC-PCR-based genotyping of the 32 *K. pneumoniae* strains with MDR phenotypes.

To get insights into the coresistance mechanism between the MDR and heavy metal tolerance, we further investigated the 32 MDR *K. pneumoniae* strains by using a phylogenetic analysis. As shown in Figure 3, the MDR strains were grouped into six clusters (Clusters A to F), wherein approximately 50.0% (16/32) fell into Cluster B, with 13 ERIC genotypes, followed by Cluster C (12.5%, 4/32) and Cluster F (12.5%, 4/32), with four ERIC genotypes. Remarkably, 84.4% (*n* = 27) of the 32 MDR strains were classified as singletons. The *K. pneumoniae* strains in *M. antiquata* were classified as more singletons (*n* = 8), followed by *E. sinensis* (*n* = 4) and *M. anguillicaudatus* (*n* = 4). Additionally, the obtained Simpson’s diversity index was 0.8488. These results provided direct evidence for the abundant genetic diversity of the MDR *K. pneumoniae* strains.

Approximately 33.0% (*n* = 31) of the 94 *K. pneumoniae* strains shared 13 ERIC genotypes. Among these, 12 strains were recovered from *C. cahayensis;* 9 from *P. clarkii*; and 10 from *B. rock cod*, *M. anguillicaudatus*, *M. antiquata*, *N. cumingi Crosse*, *H. rubra*, and *S. strictus*. Additionally, three ERIC genotypes contained the strains isolated from different species of aquatic animals. For instance, three strains sharing ERIC genotype *K. p*00023 were isolated from *H. rubra* (*K. pneumoniae* 8-2-2-3) and *M. antiquata* (*K. pneumoniae* 8-2-1-8 and 8-2-1-9). Similarly, ERIC genotype *K. p*00046 (*n* = 4) was shared by the *K. pneumoniae* 7-17-1, 7-17-2, 7-17-16, and 8-1-12-14 strains, which were isolated from *C. cahayensis* and *P. clarkii*. ERIC genotype *K. p*00057 was shared by *K. pneumoniae* 8-1-12-1 and 7-8-2 strains from *P. clarkii* and *S. strictus*, respectively. These results suggested the possible transmission of *K. pneumoniae* among the aquatic animals.

All the 32 MDR *K. pneumoniae* strains were resistant to two or more heavy metals. For instance, the isolate *K. pneumoniae* 7-5-2 from *M. anguillicaudatus* resisted six antibiotics (CHL/CIP/KAN/NOR/SXT/TET), and also tolerated five heavy metals (Cr^3+^/Cu^2+^/Hg^2+^/Pb^2+^/Zn^2+^). The isolate *K. pneumoniae* 8-2-1-11 from *M. antiquata* tolerated seven heavy metals (Cd^2+^/Cr^3+^/Cu^2+^/Hg^2+^/Mn^2+^/Pb^2+^/Zn^2+^) and also resisted three antibiotics (CHL/KAN/SXT). These results indicated close relatedness between the MDR and heavy metal tolerance of the *K. pneumoniae* strains.

### 3.8. Genome Features of the K. pneumoniae 8-2-5-4 Isolate from Serotype K2

**Figure 4:**
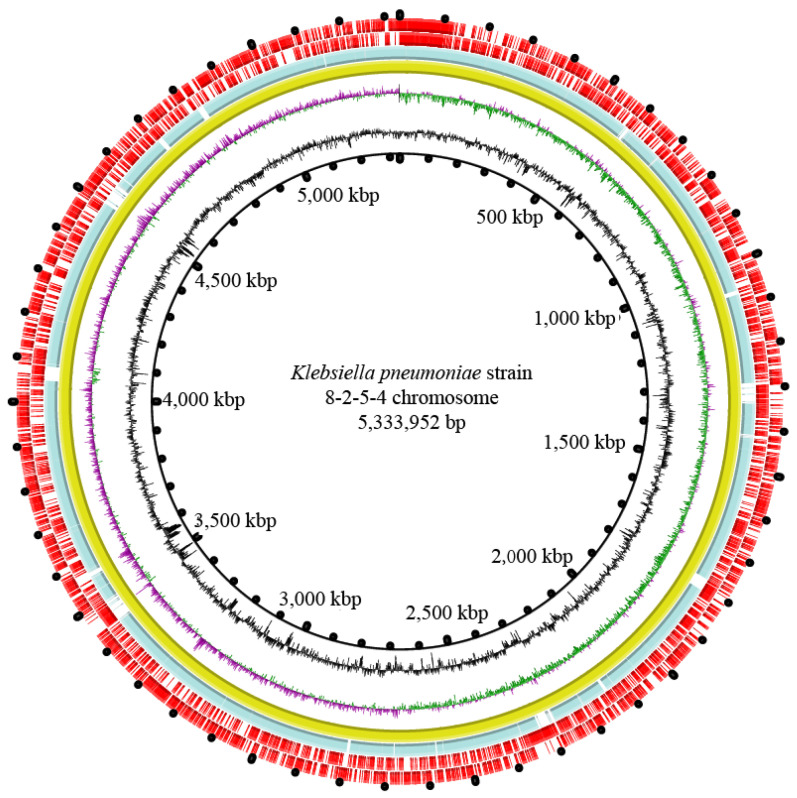
The genome circle map of *K. pneumoniae* 8-2-5-4 recovered from *T. granosa*. Circles from inside to outside were described in our recent research [48]. The reference genome was *K. pneumoniae* subsp. *pneumoniae* HS11286 (GenBank accession no. CP003200.1) (the third circle), and the draft genome sequence of *K. pneumoniae* 8-2-5-4 (the fourth circle) was determined in this study (GenBank accession no. JALJQQ000000000).

The 94 *K. pneumoniae* strains were also detected for the virulent serotypes (K1, K2, K5, K20, K54, and K57) by using the PCR assay, and the results revealed that the *K. pneumoniae* 8-2-5-4 isolate recovered from *T. granosa* tested positive for the serotype K2 gene, whereas the others tested negative for the virulent serotypes tested. Therefore, the genome sequence of this isolate was determined by using the Illumina Hiseq 10 technique. The obtained genome size of *K. pneumoniae* 8-2-5-4 (serotype K2) was 5,432,647 bp with 57.32% GC contents (Figure 4). Approximately 107,851 clean single reads were obtained and assembled into 64 scaffolds with a sequencing depth of 271.87-fold (on average). Notably, approximately 5042 protein-coding genes were annotated, of which 1230 encoded unknown proteins. MGEs were also identified, including GIs (*n* = 11), prophage gene clusters (*n* = 1), and ISs (*n* = 2) (Table 5). The draft genome sequence of *K. pneumoniae* 8-2-5-4 isolate from *T. granosa* was deposited in the GenBank database under accession number JALJQQ000000000. Additionally, the MLST analysis revealed that *K. pneumoniae* 8-2-5-4 strain from serotype K2 belonged to the ST-2026.

Approximately 89 genes involved in bacterial pathogenesis were identified in the *K. pneumoniae* 8-2-5-4 genome, which were related to adherence, antiphagocytosis, invasion, serum resistance, the iron uptake system, regulation, and the secretion system (Table S6). These results highlighted the potential virulence of *K. pneumoniae* 8-2-5-4.

*K. pneumoniae* 8-2-5-4 showed resistance to TET. Unexpectedly, approximately 36 resistance-related genes were identified in the *K. pneumoniae* 8-2-5-4 genome (Table S7), of which most genes were involved in the antibiotic efflux, antibiotic inactivation, reduced permeability to antibiotics, and antibiotic target alteration. For example, 15 genes were related to the resistance to cephalosporin and fluoroquinolone, and 12 to TET, consistent with the resistance phenotype of this bacterium.

*K. pneumoniae* 8-2-5-4 showed tolerance to Cr^3+^/Cu^2+^/Hg^2+^/Zn^2+^. Remarkably, 70 heavy metal tolerance–related genes were identified in the *K. pneumoniae* 8-2-5-4 genome (Table S8). For example, 16 genes were involved in the Cu tolerance, 13 genes in the Zn tolerance, and 19 genes in the tolerance to multiple heavy metals (e.g., Cr^3+^, Co^2+^, Cd^2+^, Hg^2+^, Ni^2+^, and Mn^2+^) (Table S8).

These results indicated the potential risk in of consuming *T. granosa* contaminated with *K. pneumoniae* 8-2-5-4.

## 4. Discussion

*K. pneumoniae* is the second leading cause of human bloodstream infections caused by Gram-negative bacteria [1,51]. *Klebsiella* species have also been identified as the third leading cause of hospital-acquired infections (HAI) in the United States [52]. Nevertheless, to the best of our knowledge, the literature on *K. pneumoniae* in aquatic environments and aquatic products is rare so far. Barati et al. [53] reported 55 *K. pneumoniae* strains isolated from aquatic environment samples collected in Perak, Malaysia [53]. Effendi et al. [54] isolated 3 *K. pneumoniae* strains from fish samples collected in East Java [54]. Recently, Håkonsholm et al. [27] reported 78 *K. pneumoniae* strains isolated from marine bivalve mollusks collected along the Norwegian coast [27]. In this study, we surveyed *K. pneumoniae* in the 41 species of aquatic animals collected in the summer of 2018 and 2019 in Shanghai, China. Of these, 39 species had no history of carrying *K. pneumoniae*. Our results revealed that *K. pneumoniae* was present in the nine species of mollusks, three species of fish, and two species of crustaceans. The results of this study also revealed the high abundance of *K. pneumoniae* in benthic animals, such as *C. cahayensis*, *M. antiquata*, and *P. clarkii*. These findings highlighted the urgent need for policy and research on the risk assessment of the edible aquatic animals for the *K. pneumoniae* contamination.

None of the 94 *K. pneumoniae* strains identified in this study carried the virulence-related genes *aerobactin*, *allS*, *iroN*, *rmpA*, *traT*, or *wcaG*. However, higher percentages of the *entB* (98.9%) and *mrkD* (85.1%) genes were detected, followed by the *fimH* (50.0%) and *ybtA* (14.9%) genes among the *K. pneumoniae* strains, which harbored nine virulence-related gene profiles. Moreover, the *K. pneumoniae* strains in the 14 species of aquatic animals had different virulence gene profiles. These results demonstrated a variable risk of potentially pathogenic *K. pneumoniae* strains in the 14 species of edible aquatic animals.

The ERIC-PCR-based fingerprinting method has been used for the relatedness or differentiation analysis of pathogenic bacteria, including *K. pneumoniae* [55]. For instance, Zhang et al. [41] reported 60 ERIC genotypes among 61 *K. pneumoniae* strains from 1200 retail foods sampled from May 2013 to April 2014 in China [41]. The ERIC-PCR conditions have been well established in our research group and applied for the genotyping of *Vibro parahaemolyticu*, *Vibrio cholerae*, and *Klebsiella oxytoca* strains isolated from various aquatic products [33,34,42]. In this study, the ERIC-PCR-based fingerprinting of the 94 *K. pneumoniae* strains revealed 76 ERIC genotypes with 63 singletons. These results demonstrated the considerable genetic diversity of the *K. pneumoniae* strains in aquatic animals.

Antimicrobial agents are widely applied in aquaculture to prevent animal diseases caused by pathogenic microorganisms [56]. However, the misuse or overuse of antibiotics comes with serious negative effects, such as the development of antibiotic resistance [57]. The emergence of MDR pathogens has become a global challenge and major burden for the treatment of infectious diseases [58]. *K. pneumoniae* is a common MDR pathogen for HAI associated with high morbidity and mortality because of the limited treatment options [59]. For example, Zhang et al. [60] reported that 7.5% of *K. pneumoniae* strains (*n* = 12) had MDR phenotypes, which were isolated from clinical samples in Wenzhou, China [60]. Fatima et al. [61] found that all *K. pneumoniae* strains (*n* = 21) were resistant to a minimum of six and a maximum of 14 antibiotics tested, which were isolated from urine (*n* = 15) and sputum (*n* = 6) samples from different patients with UTIs and respiratory tract infections (RTIs) at the Boland Medical Center in Quetta, Pakistan [61]. In this study, approximately 34.0% of the *K. pneumoniae* strains displayed MDR phenotypes. Notably, the highest rate (69.2%) of MDR strains was found in the fish. Moreover, the MDR phenotypes were predominant among *K. pneumoniae* strains in *M. anguillicaudatus* (100%), all of which were resistant to six antimicrobial agents. These results indicated higher antimicrobial exposure levels or contaminated sources in these aquatic animals.

The results of this study also revealed that the SXT resistance was predominant (52.1%) among the *K. pneumoniae* strains, one-third of which were also resistant to CHL (31.9%) and TET (27.7%). SXT belonged to the fluoroquinolone antibiotics and was frequently detected in surface water environments [62]. A high occurrence (28%) of SXT-resistant *K. pneumoniae* strains was also found in untreated hospital waste in Bangladesh [58]. Consistent with the results in this study, the higher occurrences of resistance to SXT, CHL and TET were also detected in *K*. *oxytoca* strains (*n* = 125) isolated from the 41 species of aquatic animals in our previous research [34]. In this study, certain percentages of intermediate susceptibility to CIP (18.1%) and KAN (13.8%) may suggest a potential resistance trend of these drugs. Additionally, our data also revealed different antibiotic resistance profiles among the *K. pneumoniae* strains in different types and species of aquatic animals. For example, the resistance rates of CHL, CIP, KAN, NOR, TET, and SXT were higher among the strains in *M. anguillicaudatus*, suggesting serious exposure of the aquatic animal to these antibiotic drugs. These results also highlighted the risk of the transmission of antibiotic resistance in *K. pneumoniae* to human populations via the food chain.

In recent decades, rapid industrialization, urbanization, and agricultural modernization may have led to the increased environment pollution by releasing a substantial amount of hazardous heavy metals [63]. For example, Zhao et al. [64] collected surface sediment samples in 14 typical intertidal areas across China, from July to September in 2015. They found that 11 areas were exposed to the moderate ecological risk of heavy metals in the sediment [64]. In this study, the 94 *K. pneumoniae* strains had different heavy metal–tolerance profiles. For instance, the tolerance to Cu^2+^ and Cr^3+^ were the most prevalent (96.8%, and 96.8%), followed by Zn^2+^ (91.5%), Pb^2+^ (89.4%), and Hg^2+^ (81.9%). Consistent with these results, high percentages of the tolerance to Cu^2+^ (84.8%), Pb^2+^ (80.8%), Cr^3+^ (66.4%), Zn^2+^ (66.4%), and Hg^2+^ (49.6%) were also observed among the *K. oxytoca* strains, in our previous study [34]. Copper is an essential trace element needed for the human body, but it is toxic if it exceeds the specified limit [65]. Chromium can cause skin allergies and increase the risk of lung cancer [66]. Direct or indirect exposure to Pb^2+^ leads to various health risks, such as cancer, stroke, memory problems, and neurological problems [67]. Metallic, inorganic mercury is a potent neurotoxicant [68]. In this study, the data showed that most *K. pneumoniae* strains were tolerant to Cr^3+^ (100% to 92.3%), Cu^2+^ (100% to 95.0%), Hg^2+^ (92.3% to 76.2%), Pb^2+^ (100% to 85.0%), and Zn^2+^ (95.2% to 90.0%). These results suggested that the heavy metal pollution likely occurred in the aquaculture environment of the aquatic animals.

A comparison of the genomic fingerprinting profiles of the 32 MDR *K. pneumoniae* strains further revealed a close relatedness between the MDR and heavy metal tolerance. For example, all the MDR *K. pneumoniae* strains were tolerant to two or more heavy metals. Notably, eight *K. pneumoniae* strains were resistant to six antibiotics and also tolerant to four to six heavy metals. Frequent and persistent heavy metal pollution had a profound impact on the composition and activity of the microbial community, likely cross-selected for antibiotic resistance, and vice versa [69].

The serotypes K1, K2, K5, K54, and K57 of *K. pneumoniae* strains are closely associated with invasive diseases of the bacterium [9]. K1/K2 strains are generally more virulent than the others [64]. As a causative agent of pyogenic liver abscess, K2 is also frequently reported in community-acquired pneumonia [70]. In this study, our results revealed that the *K. pneumoniae* 8-2-5-4 strain isolated from *T. granosa* belonged to the serotype K2, whereas no virulent serotypes (K1, K2, K5, K54, and K57) were found in the other 93 *K. pneumoniae* strains. Mucoviscosity-associated gene A (*magA* gene) encodes a polysaccharide polymerase enzyme that is specific to serotype K1 [71,72]. In this study, the *magA* was absent from all the *K. pneumoniae* strains.

A draft genome sequence (5,432,731 bp) of *K. pneumoniae* 8-2-5-4 (serotype K2) was determined using the Illumina Hiseq 10 technique. This strain was identified as *K. pneumoniae* ST-2026. Sequences of the ST-2026 from four *K. pneumoniae* strains, which were isolated from chicken, from Asia (unknown detailed source), were first deposited in the public database by Zhang Lifeng, National Institute for Communicable Disease Control and Prevention, Chinese Center for Disease Control and Prevention, China, on 21 September 2015 (https://cge.food.dtu.dk/services/MLST/ (accessed on 23 November 2022)). These suggested the possible transmission of *K. pneumoniae* ST-2026 strains between chicken and the aquatic animal *T*. *granosa*. Remarkably, approximately 89 virulence-related genes were identified in the *K. pneumoniae* 8-2-5-4 genome, which related mainly to adherence, antiphagocytosis, invasion, the iron uptake system, regulation, the secretion system, and serum resistance. Of these, the highest proportion (25.8%) of the virulence-related genes functioned in adhesion. Studies have shown that adherence is essential for the colonization, biofilm formation, and epithelial cell invasion of pathogenic *K. pneumoniae* [73]. In this study, comparative genomic analysis also revealed 36 antibiotic resistance–related genes in the *K. pneumoniae* 8-2-5-4 genome. For example, the *TolC* gene, which encoded an important mediator of antibiotic resistance in *K. pneumoniae* for the outer membrane efflux channel [29], was involved in the resistance to various drug classes. The *ramA* gene encoded an intrinsic regulator in *K. pneumoniae*, which played an important role in the bacterial response to antibiotic exposure [74]. The *TEM-116* and *SHV-38* genes encoded extended-spectrum β-lactamases (ESBLs) that can hydrolyze penicillins, broad-spectrum cephalosporins, and monobactams [30]. The diversity of resistance genes, gene variance, and selective pressure from the environment may result in the difference between resistance phenotypes and resistance genotypes. Meanwhile, approximately 70 heavy metal–tolerance genes were identified in the *K. pneumoniae* 8-2-5-4 genome. For example, 16 genes were involved in Cu tolerance, 13 genes in Zn tolerance, and 19 genes in the tolerance to multiple heavy metals (e.g., Cr^3+^, Co^2+^, Cd^2+^, Hg^2+^, Ni^2+^, and Mn^2+^), consistent with the Cr^3+^/Cu^2+^/Hg^2+^/Zn^2+^ phenotype of this bacterium. These results provided the genome-level evidence for the potential virulence and coresistance of *K. pneumoniae* 8-2-5-4 isolate from *T. granosa*. The pathogenesis of the bacterium should be further investigated in the future research by using cell and animal-mode techniques.

## 5. Conclusions

In this study, we for the first time investigated the prevalence, virulence, and resistance of *K. pneumoniae* in 41 commonly consumed species of aquatic animals collected in the summers of 2018 and 2019 in Shanghai, China. In these 39 species, the bacterium has never been detected. *K. pneumoniae* was found in the 14 species of aquatic animals, including *A. woodiana*, *B. areolata*, Blotchy rock cod, *C. auratus*, *C. cahayensis*, *E. sinensis*, *H*. *rubra*, *M. anguillicaudatus*, *M. antiquata*, *M. veneriformis*, *N*. *cumingi Crosse*, *P. clarkii*, *S. strictus*, and *T*. *granosa*. Except *P. clarkii*, the other 13 species had no history of carrying *K. pneumoniae*. The high abundance of *K. pneumoniae* was found in benthic animals, such as *C. cahayensis*, *M. antiquata*, and *P. clarkii*.

The higher incidence of virulence-related genes *entB* (98.9%) and *mrkD* (85.1%) was present in the 94 *K. pneumoniae* strains, followed by *fimH* (50.0%) and *ybtA* (14.9%). Conversely, the *aerobactin, allS*, *iroN*, *rmpA*, *traT*, and *wcaG* genes were absent from all the strains. Resistance to SXT (52.1%) was predominant, followed by resistance to CHL (31.9%) and TET (27.7%). Approximately 34.0% of the *K. pneumoniae* strains had MDR phenotypes. Most strains were also tolerant to Cu^2+^ (96.8%), Cr^3+^ (96.8%), Zn^2+^ (91.5%), Pb^2+^ (89.4%), and Hg^2+^ (81.9%). The 94 *K. pneumoniae* strains in the 14 species of aquatic animals had different virulence and coresistance profiles, which were classified into 76 ERIC genotypes with 63 singletons.

Additionally, the genome features of the potentially virulent *K. pneumoniae* 8-2-5-4 strain from serotype K2 (ST-2026), isolated from the mollusk *T granosa*, were characterized. Remarkably, approximately 89 virulence-, 36 antibiotic resistance–, and 70 heavy metal tolerance–related genes were identified in the *K. pneumoniae* 8-2-5-4 genome (5,432,647 bp, GenBank accession number JALJQQ000000000).

Overall, the results of this study demonstrated the considerable genetic diversity and tight coresistance relatedness of the *K. pneumoniae* strains from the 14 species of aquatic animals, and they fill gaps in the risk assessment of *K. pneumoniae* in edible aquatic animals.

## Figures and Tables

**Table 1:** The virulence-related genes in the 94 *K. pneumoniae* strains isolated from the 14 species of aquatic animals.

Aquatic Animal	Species	Percentage of Virulence-Related Gene (%)									
		*aerobactin*	*allS*	*entB*	*fimH*	*iroN*	*mrkD*	*rmpA*	*traT*	*wcaG*	*ybtA*
Mollusks	*A. woodiana*	0	0	100	0	0	0	0	0	0	0
	*B. areolata*	0	0	100	0	0	100	0	0	0	100
	*C. cahayensis*	0	0	100	96.7	0	83.3	0	0	0	16.7
	*H. rubra*	0	0	100	0	0	100	0	0	0	0
	*M. antiquata*	0	0	100	0	0	100	0	0	0	0
	*M. veneriformis*	0	0	100	50.0	0	0	0	0	0	0
	*N. cumingi Crosse*	0	0	100	0	0	0	0	0	0	0
	*S. strictus*	0	0	100	100	0	66.7	0	0	0	0
	*T. granosa*	0	0	100	33.3	0	33.3	0	0	0	0
Fish	*B. rock cod*	0	0	83.3	0	0	83.3	0	0	0	0
	*C. auratus*	0	0	100	0	0	100	0	0	0	0
	*M. anguillicaudatus*	0	0	100	100	0	100	0	0	0	0
Crustaceans	*E. sinensis*	0	0	100	100	0	100	0	0	0	0
	*P. clarkii*	0	0	100	6.7	0	100	0	0	0	53.3

**Table 2:** The virulence-related gene profiles in the 94 *K. pneumoniae* strains.

No. of Genes	Gene	No. of Strains
1	*entB^+^*	7
	*mrkD^+^*	1
2	*entB^+^/fimH^+^*	5
	*entB^+^/mrkD^+^*	29
	*entB^+^/ybtA^+^*	1
3	*entB^+^/fimH^+^/mrkD^+^*	38
	*entB^+^/mrkD^+^/ybtA^+^*	9
	*entB^+^/fimH^+^/ybtA^+^*	1
4	*entB^+^/fimH^+^/mrkD^+^/ybtA^+^*	3

**Table 3:** The resistance of the 94 *K. pneumoniae* strains to the nine antibiotics.

	Percentage of the Strains (%)								
	CHL	CIP	GEN	IPM	KAN	MEM	NOR	SXT	TET
Resistance	31.9	13.8	6.4	0.00	22.3	0	8.5	52.1	27.7
Intermediary sensitivity	1.1	18.1	9.6	1.10	13.8	0	9.6	8.5	9.6
Sensitivity	67	68.1	84	98.90	63.8	100	81.9	39.4	62.8

Note: CHL—chloramphenicol; CIP—ciprofloxacin; GEN—gentamicin; IPM—imipenem; KAN—kanamycin; MEM—meropenem; NOR—norfloxacin; SXT—sulfamethoxazole-trimethoprim; and TET—tetracycline.

**Table 4:** The antibiotic resistance profiles of the 94 *K. pneumoniae* strains in the 14 species of aquatic animals.

Aquatic Animal	Species		Percentage of Resistant Strains (%)							
		CHL	CIP	GEN	IPM	KAN	NOR	MEM	SXT	TET
Mollusks	*A. woodiana*	0	0	0	0	0	0	0	0	0
	*B. areolata*	0	0	0	0	0	0	0	100	0
	*C. cahayensis*	6.7	0	6.7	0	3.3	0	0	46.7	10
	*H. rubra*	40.0	0	20	0	40	0	0	40.0	40
	*M. antiquata*	76.9	23.1	15.4	0	61.5	0	0	84.6	46.2
	*M. veneriformis*	100	0	50	0	50	0	0	100	50
	*N. cumingi Crosse*	0	0	0	0	0	0	0	0	0
	*S. strictus*	66.7	33.3	0	0	0	33.3	0	100	66.7
	*T. granosa*	0	0	0	0	0	0	0	33.3	33.3
Fish	*B. rock cod*	33.3	33.3	0	0	33.3	0	0	50	50
	*C. auratus*	0	0	0	0	0	0	0	0	0
	*M. anguillicaudatus*	100	100	0	0	100	100	0	100	100
Crustaceans	*E. sinensis*	66.7	16.7	0	0	16.7	16.7	0	66.7	16.7
	*P. clarkii*	0	0	0	0	0	0	0	13.3	6.7

**Table 5:** Genome features of *K. pneumoniae* 8-2-5-4 isolate recovered from *T. granosa*.

Genome Feature	*K. pneumoniae* 8-2-5-4
Genome size (bp)	5,432,731
DNA G + C (%)	57.32
DNA scaffold	64
Predicted gene	5143
Protein-coding gene	5042
RNAs gene	224
Genes assigned to COG	4777
Genes with unknown function	1230
Genomic island	11
Prophage gene cluster	1
Integron	0
Insertion sequence	2

## Data Availability

The draft genome sequence of *K. pneumoniae* 8-2-5-4 isolate recovered from *T. granosa* was deposited in the GenBank database under accession number JALJQQ000000000.

## Supplementary Materials

The following supporting information can be downloaded at https://www.mdpi.com/article/10.3390/foods11244058/s1: Table S1—The 41 species of aquatic animals and the isolated *K. pneumoniae* strains; Table S2—The oligonucleotide primers used in this study [75,76]; Table S3—The sequenced 16S rRNA and virulence-related genes in the representative *K. pneumoniae* strains; Table S4—The distribution of the *K. pneumoniae* strains in the aquatic animal samples originating from different regions and fish markets in China; Table S5—Heavy metal tolerance of the 94 *K. pneumoniae* strains evaluated in this study; Table S6—The predicted virulence-related genes in the *K. pneumoniae* 8-2-5-4 genome; Table S7—The predicted antibiotic resistance–related genes in the *K. pneumoniae* 8-2-5-4 genome; Table S8—The prediction heavy metal resistance–related genes in the *K. pneumoniae* 8-2-5-4 genome.
