# Supplementary material for: First Report of Potentially Pathogenic Klebsiella pneumoniae from Serotype K2 in Mollusk Tegillarca granosa and Genetic Diversity of Klebsiella pneumoniae in 14 Species of Edible Aquatic Animals
Source: Foods. 2022 Dec 15;11(24):4058. doi: 10.3390/foods11244058 (PMC9778296; doi:10.3390/foods11244058)
Supplement: Supplementary file 1 [file foods-11-04058-s001.zip › foods-1977138-supplementary.pdf]

Supplementary Materials

Table S1. The 41 species of aquatic animals and the isolated *K. pneumoniae* strains.

Aquatic Product	Species	Common Name	No. of Isolates
Fish	*Aristichthys nobilis*	Variegated carp	0
	Blotchy rock cod	Grouper	6
	Carassius auratus	Crucian	1
	Carassius auratus	Ditrema temmincki Bleeker	0
	Channa argus	Snakeheaded fish	0
	Erythroculter ilishaeformis	Topmouth culter	0
	Lctalurus punctatus	Channel catfish	0
	Lateolabrax japonicus	Perch	0
	Misgurnus anguillicaudatus	Loach	6
	Mylopharyngodon piceus	Black carp	0
	Nibea albiflora	Spotted maigre	0
	Parabramis pekinensis	Bream	0
	Pelteobagrus fulvidraco	Banded catfish	0
	Plectorhynchus cinctus	Grunt	0
	Scophthalmus maximus	Turbot	0
	Sparus berda	Yellowfin snapper	0
	Siniperca chuatsi	Mandarin fish	0
Mollusks	*Anodonta woodiana*	Unionidae	1
	Antigona lamellaris	Diagonal clam	0
	Atrina pectinata	Comb pen shell	0
	Azumapecten farreri	Farrer's scallop	0
	Babylonia areolata	Cowrie shell	1
	Babylonia formosae habei	Ivory shell	0
	Cipangopaludina cahayensis	Mudsnail	30
	Haliotis rubra	Abalone	5
	Mactra antiquata	Mussel	13
	Mactra veneriformis	White clam seed	2
	Moerella iridescens	Sea-pumpkin seeds	0
	Mytilus edulis	Mussel	0
	Neptunea cumingi Crosse	Sea snail	2
	Ostrea gigas thunberg	Oyster	0
	Pseudocardium sachalinense	Arctic surf clam	0
	Saxidomus purpuratus	Sowerby	0
	Scapharca subcrenata	Ark shell	0
	Sinonovacula constricta	Razor clam	0
	Solen strictus	Razor clam	3
	Spisula solidissima	Golden scallop	0
	Tegillarca granosa	Blood cockle	3
Crustaceans	*Eriocheir sinensis*	Chinese mitten crab	6
	Penaeus vannamei	Whiteshrimp	0
	Procambarus clarkii	Crayfish	15

Table S2. The oligonucleotide primers used in this study.

Gene	Primer	Sequence（5′->3′）	Amplicon Size (bp)	Elongation Time (min)	Annealing Temperature (°C)	Reference
16S rRNA	27-F	AGAGTTTGATCCTGGCTCAG	1540	2	52	[75]
	1429-R	TACGGCTACCTTGTTACGAC				
aerobactin	*aerobactin*-F	GTGCTGTCGATGAGCGATGC	944	1	55	[36]
	aerobactin-R	GTGAGCCAGGTTTCAGCGTC				
alls	*alls*-F	CATTACGCACCTTTGTCAGC	764	1	60	[76]
	alls-R	GAATGTGTCGGCGATCAGCTT				
entB	*entB*-F	ATTTCCTCAACTTCTGGGGC	371	1	57	[37]
	entB-R	AGCATCGGTGGCGGTGGTCA				
fimH	*fimH*-F	TACTGCTGATGGGCTGGTC	640	1	50	[37]
	fimH-R	GCCGGAGAGGTAATACCCC				
iroN	*iroN*-F	AAGTCAAAGCAGGGGTTGCCCG	665	1	63	[38]
	iroN-R	GACGCCGACATTAAGACGCAG				
magA (K1)	*magA*-F	GGTGCTCTTTACATCATTGC	1283	2	59	[39]
	magA-R	GCAATGGCCATTTGCGTTAG				
mrkD	*mrkD*-F	CCACCAACTATTCCCTCGAA	240	1	60	[40]
	mrkD-R	ATGGAACCCACATCGACATT				
rmpA	*rmpA*-F	ACTGGGCTACCTCTGCTTCA	535	1	61	[15]
	rmpA-R	CTTGCATGAGCCATCTTTCA				
traT	*traT*-F	GGTGTGGTGCGATGAGCACAG	290	1	63	[38]
	traT-R	CACGGTTCAGCCATCCCTGAG				
wcaG	*wcaG*-F	GGTTGGTCAGCAATCGTA	169	1	57	[9]
	wcaG-R	ACTATTCCGCCAACTTTTGC				
ybtA	*ybtA*-F	ATGACGGAGTCACCGCAAAC	950	1	53	[41]
	ybtA-R	TTACATCACGCGTTTAAAGG				
K2	K2-F	GACCCGATATTCATACTTGACAGAG	641	1	58	[9]
	K2-R	CCTGAAGTAAAATCGTAAATAGATGGC				
K5	K5-F	TGGTAGTGATGCTCGCGA	280	1	60	[9]
	K5-R	CCTGAACCCACCCCAATC				
K20	K20-F	CGGTGCTACAGTGCATCATT	741	1	58	[9]
	K20-R	GTTATACGATGCTCAGTCGC				
K54	K54-F	CATTAGCTCAGTGGTTGGCT	881	1	62	[9]
	K54-R	GCTTGACAAACACCATAGCAG				
K57	K57-F	CTCAGGGCTAGAAGTGTCAT	1037	2	61	[9]
	K57-R	CACTAACCCAGAAAGTCGAG				
ERIC	ERIC1R	ATGTAAGCTCCTGGGGATTCAC				[42]
	ERIC2	AAGTAAGTGACTGGGGTGAGCG				

Table S3. The sequenced 16S rRNA and virulence-related genes in the representative *K. pneumoniae* isolates.

K. pneumoniae Isolate	Gene	NCBI Accession Number
7-5-1	16S rRNA	OM816744
7-5-4	16S rRNA	OM816745
7-10-14	16S rRNA	OM816746
7-13-2	16S rRNA	OM816747
7-17-8	16S rRNA	OM816748
7-17-16	16S rRNA	OM816749
8-1-12-1	16S rRNA	OM816750
8-1-12-5	16S rRNA	OM816751
8-2-5-4	16S rRNA	OM816752
8-2-10-5	16S rRNA	OM816753
8-2-2-22	*entB*	OK546015
8-2-2-14	*entB*	OK546016
8-1-7-4	*entB*	OK546018
8-1-12-1	*entB*	OK576466
8-1-12-13	*entB*	OK576467
7-5-2	*entB*	OK576468
7-17-21	*entB*	OK576469
7-17-2	*entB*	OK576470
7-17-1	*entB*	OK576471
7-17-3	*entB*	OK576472
7-17-1	*fimH*	MZ366459
7-17-2	*mrkD*	MZ380316
7-17-6	*ybtA*	MZ380315
8-1-7-4	*ytbA*	OK546017

Table S4. The distribution of the *K. pneumoniae* strains in the aquatic animal samples originating from different regions and fish markets in China.

Province/City	Species	Fish Market	No. of strain	Percentage (%)
Fujian	*B. areolata, N. cumingi Crosse, S. strictus, T. granosa*	Luchao Port Aquatic Market	8	8.51
Hunan	*M. antiquata*	Jiangyang Aquatic Market	13	13.83
Jiangsu	*A. woodiana, H. rubra*	Jiangyang Aquatic Market	6	6.38
	M. veneriformis	Luchao Port Aquatic Market	2	2.13
Shandong	*C. cahayensis*	Luchao Port Aquatic Market	30	31.91
Shanghai	*P. clarkii*	Jiangyang Aquatic Market	15	15.96
	E. sinensis	Luchao Port Aquatic Market	6	6.38
Zhejiang	*B. rock cod, T. granosa*	Jiangyang Aquatic Market	7	7.45
Zhejiang	*C. auratus, M. anguillicaudatus*	Luchao Port Aquatic Market	7	7.45

Table S5. Heavy metal tolerance of the 94 *K. pneumoniae* strains evaluated in this study.

Heavy metal	Number of isolates with a maximum observed MIC (μg/mL)											Resistance	
	3.125	6.25	12.5	25	50	100	200	400	800	1600	3200	No.	(%)
Cd^{2+}					2	6	55 [a]	28	3			31	33.0
Cr^{3+}								1	2 [a]	55	36	91	96.8
Cu^{2+}								3 [a]	14	74	3	91	96.8
Hg^{2+}	3	14 [a]	43	27	7							77	81.9
Mn^{2+}							1	3	19	57 [a]	14	14	14.9
Ni^{2+}							20	43	31 [a]			0	0
Pb^{2+}							1	1	8 [a]	8	76	84	89.4
Zn^{2+}							8 [a]	66	19	1	0	86	91.5

Note: a, the minimal inhibition concentration (MIC) of the quality control strain *E. coli* K12.

Table S6. The **p**redicted virulence-related genes in the *K. pneumoniae* 8-2-5-4 genome.

Gene ID	Location	VFDB ID	Virulence Factor	Description
gene4612	Scaffold22	VFG044322	Sal	A major trigger of inflammation and bacterial dissemination induced during *K. pneuminiae* lung infection
gene1449	Scaffold3	VFG048237	Type I fimbriae	Adhering to human mucosal or epithelial surfaces
gene1450	Scaffold3	VFG048247		
gene1453	Scaffold3	VFG048277		
gene1448	Scaffold3	VFG048229		
gene1451	Scaffold3	VFG048257		
gene1455	Scaffold3	VFG048297		
gene1456	Scaffold3	VFG048307		
gene1452	Scaffold3	VFG048267		
gene1457	Scaffold3	VFG048317		
gene1454	Scaffold3	VFG048287		
gene3582	Scaffold11	VFG048784	T6SS	Antibacterial activity
gene3588	Scaffold11	VFG048740		
gene5049	Scaffold35	VFG048683		
gene5143	Scaffold60	VFG048639		
gene3583	Scaffold11	VFG048776		
gene5047	Scaffold35	VFG048661		
gene5054	Scaffold35	VFG048732		
gene3584	Scaffold11	VFG048767		
gene5050	Scaffold35	VFG048693		
gene5147	Scaffold66	VFG048628		
gene5046	Scaffold35	VFG048650		
gene5144	Scaffold61	VFG048639		
gene3585	Scaffold11	VFG048758		
gene5051	Scaffold35	VFG048704		
gene5055	Scaffold35	VFG048723		
gene5056	Scaffold35	VFG048723		
gene3586	Scaffold11	VFG048751		

Gene ID	Location	VFDB ID	Virulence Factor	Description
gene0576	Scaffold1	VFG048683		
gene3589	Scaffold11	VFG048736		
gene5053	Scaffold35	VFG048723		
gene0261	Scaffold1	VFG048797	Capsule	Assisting in evading the host immune system by protecting bacteria from opsonophagocytosis and serum killing
gene0262	Scaffold1	VFG048808		
gene0277	Scaffold1	VFG048990		
gene0264	Scaffold1	VFG048830		
gene0263	Scaffold1	VFG048819		
gene0275	Scaffold1	VFG048980		
gene0276	Scaffold1	VFG048985		
gene0274	Scaffold1	VFG048972		
gene4125	Scaffold16	VFG048606	Aerobactin	Citrate-hydroxamate siderophore aerobactin is needed for successful infection by hypervirulent *K. pneumoniae* in pneumonic infections
gene4123	Scaffold16	VFG048600		
gene4126	Scaffold16	VFG048609		
gene4124	Scaffold16	VFG048603		
gene4127	Scaffold16	VFG048621		
gene3400	Scaffold10	VFG001443	OmpA	Contributes to brain microvascular endothelial cells (BMECs) invasion via a ligand-receptor interaction. Interaction of OmpA with its receptor induces actin condensation at the binding site. The actin reorganization induced by *E. coli* depends on activation of several host proteins involved in signaling, including focal adhesion kinase, PI3-kinase, PKC, and caveolin-1.OmpA also binds to C4b-binding protein, to avoid complement-mediated attack
gene0606	Scaffold2	VFG013465	LOS	Major immunogen.LOS phosphorylcholine (ChoP) may influence invasion via interaction with PAF receptor and stimulates of inflammatory signals.LPS phase variation is characterized by the spontaneous loss and gain of oligosaccharide structures present in the outer core. the phase variable expression of LPS biosynthesis genes promotes evasion of antigen-specific host immune defences and allow colonization of different host microenvironments
gene1548	Scaffold4	VFG048488	Ent	May be necessary for penetration to deeper tissues
gene1551	Scaffold4	VFG048459		
gene1544	Scaffold4	VFG048518		
gene1555	Scaffold4	VFG048429		
gene1549	Scaffold4	VFG048478		
gene1550	Scaffold4	VFG048468		

Gene ID	Location	VFDB ID	Virulence Factor	Description
gene1556	Scaffold4	VFG048419		
gene1547	Scaffold4	VFG048498		
gene1554	Scaffold4	VFG048439		
gene1553	Scaffold4	VFG048449		
gene1557	Scaffold4	VFG048409		
gene1545	Scaffold4	VFG048508		
gene1543	Scaffold4	VFG048528		
gene2594	Scaffold7	VFG049133	AcrAB	May mediate resistance against host-derived antimicrobial peptides; associated with antibiotic resistance
gene2595	Scaffold7	VFG049144		
gene2527	Scaffold6	VFG049144		
gene1436	Scaffold3	VFG048327	Type 3 fimbriae	Mediating the formation of biofilms on biotic and abiotic surfaces
gene1439	Scaffold3	VFG043623		
gene1441	Scaffold3	VFG043625		
gene1440	Scaffold3	VFG043624		
gene1438	Scaffold3	VFG048348		
gene1442	Scaffold3	VFG043626		
gene1437	Scaffold3	VFG048338		
gene1443	Scaffold3	VFG043627		
gene0400	Scaffold1	VFG049018	RcsAB	RcsB combined with the unstable auxiliary regulator RcsA to bind to an RcsAB box in the promoter region to upregulate the cps genes expression
gene0156	Scaffold1	VFG049007		
gene0258	Scaffold1	VFG049084	LPS	Resistant to serum complement.Also play a role in protecting bacteria from antimicro-bial peptides, including polymyxin antibiotics
gene0259	Scaffold1	VFG049095		
gene0254	Scaffold1	VFG049040		
gene0255	Scaffold1	VFG049051		
gene0256	Scaffold1	VFG049062		
gene0253	Scaffold1	VFG049029		
gene0257	Scaffold1	VFG049073		
gene4892	Scaffold28	VFG046465	EF-Tu	Surface-expressed elongation factor-Tu (EF-Tu) mediates attachment by interacting with host cell nucleolin
gene4949	Scaffold31	VFG046465		
gene2211	Scaffold5	VFG046465		
gene5098	Scaffold42	VFG046465		

Note: VFDB, The virulence factor database (http://www.mgc.ac.cn/VFs/main.htm).

Table S7. The **p**redicted antibiotic resistance-related genes in the *K. pneumoniae* 8-2-5-4 genome.

Gene ID	Location	Gene	CARD ID	Description	Drug Class	Resistance Mechanism
gene0291	Scaffold1	*mdtC*	3000794	MdtC is a transporter that forms a heteromultimer complex with MdtB to form a multidrug transporter. MdtBC is part of the MdtABC-TolC efflux complex. In the absence of MdtB, MdtC can form a homomultimer complex that results in a functioning efflux complex with a narrower drug specificity. mdtC corresponds to 3 loci in Pseudomonas aeruginosa PAO1 (gene name: muxC/muxB) and 3 loci in Pseudomonas aeruginosa LESB58.	Aminocoumarin	Antibiotic efflux
gene0290	Scaffold1	*mdtB*	3000793	MdtB is a transporter that forms a heteromultimer complex with MdtC to form a multidrug transporter. MdtBC is part of the MdtABC-TolC efflux complex.	Aminocoumarin	Antibiotic efflux
gene0294	Scaffold1	*baeR*	3000828	BaeR is a response regulator that promotes the expression of MdtABC and AcrD efflux complexes.	Aminocoumarin; aminogly-coside	Antibiotic efflux
gene0194	Scaffold1	*MdtK*	3001327	A multidrug and toxic compound extrusions (MATE) transporter conferring resistance to norfloxacin, doxorubicin and acriflavine.	Fluoroqui-nolone	Antibiotic efflux
gene0394	Scaffold1	*YojI*	3003952	YojI mediates resistance to the peptide antibiotic microcin J25 when it is expressed from a multicopy vector. YojI is capable of pumping out microcin molecules. The outer membrane protein TolC in addition to YojI is required for export of microcin J25 out of the cell. Microcin J25 is thus the first known substrate for YojI.	Peptide	Antibiotic efflux
gene0261	Scaffold1	*ugd*	3003577	PmrE is required for the synthesis and transfer of 4-amino-4-deoxy-L-arabinose (Ara4N) to Lipid A, which allows gram-negative bacteria to resist the antimicrobial activity of cationic antimicrobial peptides and antibiotics such as polymyxin	Peptide	Antibiotic target alteration

Gene ID	Location	Gene	CARD ID	Description	Drug Class	Resistance Mechanism
gene0635	Scaffold2	*H-NS*	3000676	H-NS is a histone-like protein involved in global gene regulation in Gram-negative bacteria. It is a repressor of the membrane fusion protein genes acrE, mdtE, and emrK as well as nearby genes of many RND-type multidrug exporters.	Cephalos-porin; cephamyc-in; fluoroqui-nolone; macrolide; penam; tetracycline	Antibiotic efflux
gene1184	Scaffold3	*K. pneumoniae KpnG*	3004588	KpnG consists of ~390 residues and resembles EmrA of *E. coli*. Disruption of the pump components KpnG-KpnH signficantly decrease resistance to azithromycin, ceftazidime, ciprofloxacin, ertapenem, erythromycin, gentamicin, imipenem, ticarcillin, norfloxacin, polymyxin-B, piperacillin, spectinomycin, tobramycin, and streptomycin	Aminoglycoside; carbapenem; cephalosporin; fluoroquinolone; macrolide; penam; penem; peptide	Antibiotic efflux
gene1185	Scaffold3	*K. pneumoniae KpnH*	3004597	KpnH consists of ~511 residues, resembles EmrB of *E. coli*, and is probably a translocase in the KpnGH-TolC efflux protein in *K. pneumoniae*. Disruption of the pump components KpnG-KpnH signficantly decrease resistance to azithromycin, ceftazidime, ciprofloxacin, ertapenem, erythromycin, gentamicin, imipenem, ticarcillin, norfloxacin, polymyxin-B, piperacillin, spectinomycin, tobramycin, and streptomycin.	Aminoglycoside; carbapenem; cephalosporin; fluoroquinolone; macrolide; penam; penem; peptide	Antibiotic efflux
gene1140	Scaffold3	*oqxA*	3003922	RND efflux pump conferring resistance to fluoroquinolone	Diaminopyrimidine; fluoroquinolone; glycylcycline; nitrofuran; tetracycline	Antibiotic efflux
gene1141	Scaffold3	*oqxB*	3003923	RND efflux pump conferring resistance to fluoroquinolone	Diaminopyrimidine; fluoroquinolone; glycylcycline; nitrofuran; tetracycline	Antibiotic efflux
gene1183	Scaffold3	*emrR*	3000516	EmrR is a negative regulator for the EmrAB-TolC multidrug efflux pump in *E. coli*. Mutations lead to EmrAB-TolC overexpression.	Fluoroquinolone	Antibiotic efflux

Gene ID	Location	Gene	CARD ID	Description	Drug Class	Resistance Mechanism
gene1659	Scaffold4	*kdpE*	3003841	kdpE is a transcriptional activator that is part of the two-component system KdpD/KdpE that is studied for its regulatory role in potassium transport and has been identified as an adaptive regulator involved in the virulence and intracellular survival of pathogenic bacteria. kdpE regulates a range of virulence loci through direct promoter binding.	Aminoglycoside	Antibiotic efflux
gene1821	Scaffold4	*E. coli mdfA*	3001328	Multidrug efflux pump in *E. coli*. This multidrug efflux system was originally identified as the Cmr/CmlA chloramphenicol exporter.	Benzalkonium chloride; rhodamine; tetracycline	Antibiotic efflux
gene2192	Scaffold5	*CRP*	3000518	CRP is a global regulator that represses MdtEF multidrug efflux pump expression.	Fluoroquinolone macrolide; penam	Aantibiotic efflux
gene2088	Scaffold5	*PmrF*	3003578	PmrF is required for the synthesis and transfer of 4-amino-4-deoxy-L-arabinose (Ara4N) to Lipid A, which allows gram-negative bacteria to resist the antimicrobial activity of cationic antimicrobial peptides and antibiotics such as polymyxin. pmrF corresponds to 1 locus in Pseudomonas aeruginosa PAO1 and 1 locus in Pseudomonas aeruginosa LESB58.	Peptide	Antibiotic target alteration
gene2306	Scaffold6	*TolC*	3000237	TolC is a protein subunit of many multidrug efflux complexes in Gram negative bacteria. It is an outer membrane efflux protein and is constitutively open. Regulation of efflux activity is often at its periplasmic entrance by other components of the efflux complex.	Aminocoumarin; aminoglycoside; carbapenem; cephalosporin; cephamycin; fluoroquinolone; glycylcycline; macrolide; penam; penem; peptide; phenicol; rifamycin; tetracycline; triclosan	Antibiotic efflux
gene2527	Scaffold6	*AcrF*	3000502	AcrF is a inner membrane transporter, similar to AcrB.	Cephalosporin; cephamycin; fluoroquinolone; penam	Antibiotic efflux
gene2319	Scaffold6	*bacA*	3002986	The bacA gene product (BacA) recycles undecaprenyl pyrophosphate during cell wall biosynthesis which confers resistance to bacitracin.	Peptide	Antibiotic target alteration

Gene ID	Location	Gene	CARD ID	Description	Drug Class	Resistance Mechanism
gene2594	Scaffold7	*K. pneumoniae acrA*	3004041	AcrA is a subunit of the AcrAB multidrug efflux system that in *K. pneumoniae*, which is encoded by the acrRAB operon.	Cephalosporin; fluoroquinolone; glycylcycline; penam; phenicol; rifamycin; tetracycline; triclosan	Antibiotic efflux
gene2595	Scaffold7	*acrB*	3000216	Protein subunit of AcrA-AcrB-TolC multidrug efflux complex. AcrB functions as a herterotrimer which forms the inner membrane component and is primarily responsible for substrate recognition and energy transduction by acting as a drug/proton antiporter.	Cephalosporin; fluoroquinolone; glycylcycline; penam; phenicol; rifamycin; tetracycline; triclosan	Antibiotic efflux
gene2722	Scaffold7	*E. coli ampH β-lactamase*	3004612	AmpH is a class C ampC-like β-lactamase and penicillin-binding protein identified in *E. coli*.	Cephalosporin; penam	Antibiotic inactivation
gene3322	Scaffold10	*mdtH*	3001216	Multidrug resistance protein MdtH	Fluoroquinolone	Antibiotic efflux
gene3331	Scaffold10	*mdtG*	3001329	The MdtG protein, also named YceE, appears to be a member of the major facilitator superfamily of transporters, and it has been reported, when overexpressed, to increase fosfomycin and deoxycholate resistances. mdtG is a member of the marA-soxS-rob regulon.	Fosfomycin	Antibiotic efflux
gene3441	Scaffold10	*msbA*	3003950	MsbA is a multidrug resistance transporter homolog from *E. coli* and belongs to a superfamily of transporters that contain an adenosine triphosphate (ATP) binding cassette (ABC) which is also called a nucleotide-binding domain (NBD). MsbA is a member of the MDR-ABC transporter group by sequence homology. MsbA transports lipid A, a major component of the bacterial outer cell membrane, and is the only bacterial ABC transporter that is essential for cell viability.	Nitroimidazole	Antibiotic efflux

Gene ID	Location	Gene	CARD ID	Description	Drug Class	Resistance Mechanism
gene3555	Scaffold11	*K. pneumoniae OmpK37*	3004122	*K. pneumoniae* outer membrane porin protein. Is preferentially detected in porin-deficient strains. Functional characterization of this new porin revealed a narrower pore than those of porins OmpK35 and OmpK36, which did not allow penetration by certain β-lactams. Also, when a resistant strain expresses porin OmpK37 is less susceptible to cefotaxime and cefoxitin than when it is expressing either OmpK36 or OmpK35.	Carbapenem; cephalosporin; cephamycin; monobactam; penam; penem	Reduced permeability to antibiotic
gene3602	Scaffold12	*FosA6*	3004111	fosA6 is a plasmid-encoded enzyme that confers resistance to fosfomycin in *E. coli* by breaking the epoxide ring of the molecule.	Fosfomycin	Antibiotic inactivation
gene3912	Scaffold14	*acrD*	3000491	AcrD is an aminoglycoside efflux pump expressed in *E. coli*. Its expression can be induced by indole, and is regulated by baeRS and cpxAR.	Aminoglycoside	Antibiotic efflux
gene4290	Scaffold18	*cpxA*	3000830	CpxA is a membrane-localized sensor kinase that is activated by envelope stress. It starts a kinase cascade that activates CpxR, which promotes efflux complex expression.	Aminocoumarin; aminoglycoside	Antibiotic efflux
gene4696	Scaffold23	*marA*	3000263	In the presence of antibiotic stress, *E. coli* overexpresses the global activator protein MarA, which besides inducing MDR efflux pump AcrAB, also down- regulates synthesis of the porin OmpF.	Carbapenem; cephalosporin; cephamycin; fluoroquinolone; glycylcycline; monobactam; penam;penem; phenicol; rifamycin; tetracycline; triclosan	Antibiotic efflux; reduced permeability to antibiotic
gene4676	Scaffold23	*SHV-38*	3001096	SHV-38 is an extended-spectrum β-lactamase found in *K. pneumoniae*.	Carbapenem; cephalosporin; penam	Antibiotic inactivation
gene4731	Scaffold24	*ramA*	3000823	RamA (resistance antibiotic multiple) is a positive regulator of AcrAB-TolC and leads to high level multidrug resistance in *K. pneumoniae*, Salmonella enterica, and Enterobacter aerugenes, increasing the expression of both the mar operon as well as AcrAB. RamA also decreases OmpF expression.	Carbapenem; cephalosporin; cephamycin; fluoroquinolone; glycylcycline; monobactam; penam; penem; phenicol; rifamycin; tetracycline; triclosan	Antibiotic efflux;reduced permeability to antibiotic

Gene ID	Location	Gene	CARD ID	Description	Drug Class	Resistance Mechanism
gene4816	Scaffold26	*K. pneumoniae KpnF*	3004583	KpnF subunit of KpnEF resembles EbrAB from *E. coli*. Mutation in KpnEF resulted in increased susceptibility to cefepime, ceftriaxon, colistin, erythromycin, rifampin, tetracycline, and streptomycin as well as enhanced sensitivity toward sodium dodecyl sulfate, deoxycholate, dyes, benzalkonium chloride, chlorhexidine, and triclosan.	Aminoglycoside; cephalosporin; macrolide; peptidec; rifamycin; tetracycline	Antibiotic efflux
gene4815	Scaffold26	*K. pneumoniae KpnE*	3004580	KpnE subunit of KpnEF resembles EbrAB from *E. coli*. Mutation in KpnEF resulted in increased susceptibility to cefepime, ceftriaxon, colistin, erythromycin, rifampin, tetracycline, and streptomycin as well as enhanced sensitivity toward sodium dodecyl sulfate, deoxycholate, dyes, benzalkonium chloride, chlorhexidine, and triclosan	Aminoglycoside; cephalosporin; macrolide; peptide; rifamycin; tetracycline	Antibiotic efflux
gene5112	Scaffold44	*tet(A)*	3000165	TetA is a tetracycline efflux pump found in many species of Gram-negative bacteria.	Tetracycline	Antibiotic efflux
gene5135	Scaffold53	*TEM-116*	3000979	TEM-116 is a broad-spectrum β-lactamase found in many species of bacteria.	Cephalosporin; monobactam; penam; penem	Antibiotic inactivation

Note: CARD, The comprehensive antibiotic resistance database (http://arpcard.Mcmaster.ca).

Table S8. The prediction heavy metal resistance-related genes in the *K. pneumoniae* 8-2-5-4 genome.

Gene ID	Location	BacMet ID	Gene	Description	Resistance to Heavy Metal
gene0066	Scaffold1	BAC0252	*mntP/yebN*	MntP; Functions as a manganese efflux pump	Mn, Mg
gene0108	Scaffold1	BAC0465	*znuC/yebM*	Zinc import ATP-binding protein ZnuC; part of the ABC transporter complex ZnuABC involved in zinc import. Responsible for energy coupling to the transport system. Transcriptionally repressed by zur (zinc uptake regulator), in response to high extracellular zinc concentrations	Zn
gene0109	Scaffold1	BAC0464	*znuB/yebI*	High-affinity zinc uptake system membrane protein ZnuB; Involved in the high-affinity zinc uptake transport system; located in cell inner membrane	Zn
gene0290	Scaffold1	BAC0646	*mdtB*	Multidrug resistance protein MdtB. Part of a tripartite efflux system composed of MdtA, MdtB and MdtC. MdtB forms a heteromultimer with MdtC. MdtABC multidrug efflux systems has a role in metal resistance.	Zn
gene0291	Scaffold1	BAC0647	*mdtC*	Multidrug resistance protein MdtC. Part of a tripartite efflux system composed of MdtA, MdtB and MdtC. MdtB forms a heteromultimer with MdtC. MdtABC multidrug efflux systems has a role in metal resistance.	Zn
gene0294	Scaffold1	BAC0596	*baeR*	Member of the two-component regulatory system BaeS/BaeR. Activates BaeR by phosphorylation which then activates the mdtABCD and probably the casABCDE-ygbT-ygbF operons.	Cu, Zn, W
gene0595	Scaffold2	BAC0434	*ychH*	Stress protein YchH; response to Hydrogen Peroxide (H2O2), cadmium; plays a role in biofilm formation as well	Cd
gene0846	Scaffold2	BAC0707	*sodB*	Fe-dependent superoxide dismutase. Destroys superoxide anion radicals which are normally produced within the cells and which are toxic to biological systems.	Se
gene1252	Scaffold3	BAC0349	*sitA*	Iron/Mn transport protein, periplasmic-binding protein SitA; sitA gene encodes a periplasmic binding protein	Mn, Fe
gene1253	Scaffold3	BAC0350	*sitB*	sitB encodes an ATP-binding protein	Mn, Fe
gene1254	Scaffold3	BAC0351	*sitC*	sitC encodes a permease (integral membrane protein); part of the sitABCD transport system	Mn, Fe
gene1467	Scaffold3	BAC0031	*arsB*	Arsenic pump membrane protein, located in cell inner membrane. Involved in arsenite and antimonite resistance. Thought to form a channel of an arsenite pump.	As, Sb
gene1628	Scaffold4	BAC0115	*cutE/lnt*	Apolipoprotein N-acyltransferas, it attaches a fatty acid to apo-nlpE (cutF)	Cu
gene1629	Scaffold4	BAC0088	*corC*	Magnesium and cobalt efflux protein CorC; Plays a role in the transport of magnesium and cobalt ions	Co, Mg
gene1714	Scaffold4	BAC0608	*modE*	Transcriptional regulator ModE. The ModE-Mo complex acts as a repressor of the modABC operon, involved in the transport of molybdate. It controls tungstate and molybdate homeostasis in bacteria.	W, Mo
gene1716	Scaffold4	BAC0609	*modA*	Molybdate-binding periplasmic protein. Involved in the transport of molybdenum into the cell. Binds molybdate with high specificity and affinity.	W, Mo
gene1717	Scaffold4	BAC0610	*modB*	Molybdenum transport system permease protein ModB. Part of the binding-protein-dependent transport system for molybdenum; probably responsible for the translocation of the substrate across the membrane.	W, Mo

Gene ID	Location	BacMet ID	Gene	Description	Resistance to Heavy Metal
gene1792	Scaffold4	BAC0253	*mntR*	Manganese transport regulator mntR; In the presence of manganese, represses expression of mntH and mntS. Up-regulates expression of mntP	Mn, Mg
gene1829	Scaffold4	BAC0540	*nfsA*	A major oxygen-insensitive nitroreductase of Escherichia coli, is a flavoprotein that is able to reduce chromate to less soluble and less toxic Cr (III). Reduction of nitroaromatic compounds using NADH. Reduces nitrofurazone by a ping-pong bi-bi mechanism possibly to generate a two-electron transfer product. Major component of the oxygen-insensitive nitroreductase activity in *E. coli*.	Cr
gene1927	Scaffold5	BAC0181	*glpF*	Glycerol uptake facilitator protein GlpF; Transporter of glycerol across the cytoplasmic membrane, with limited permeability to water and small uncharged compounds such as polyols	As, Sb
gene2076	Scaffold5	BAC0312	*pitA*	Low-affinity inorganic phosphate transporter 1, PitA; Can also transport arsenate	Zn, Te
gene2082	Scaffold5	BAC0272	*nikC*	Nickel transport system permease protein NikC; Transmembrane protein; Involved in a nickel transport system, probably translocates nickel through the bacterial inner membrane. Part of the Nik operon (NikABCDE)	Ni
gene2083	Scaffold5	BAC0271	*nikB*	Nickel transport system permease protein NikB; Transmembrane protein; Involved in a nickel transport system, probably translocates nickel through the bacterial inner membrane. Part of the Nik operon (NikABCDE)	Ni
gene2310	Scaffold6	BAC0469	*zupT/ygiE*	Zinc transporter ZupT; Mediates zinc uptake. May also transport other divalent cations such as copper and cadmium ions.	Zn, Fe, Co, Ni, Cu, Cd
gene2343	Scaffold6	BAC0181	*glpF*	Glycerol uptake facilitator protein GlpF; Transporter of glycerol across the cytoplasmic membrane, with limited permeability to water and small uncharged compounds such as polyols	As, Sb
gene2569	Scaffold7	BAC0166	*fetB/ybbM*	FetB/YbbM is a membrane protein. FetAB allows for increased resistance to oxidative stress in the presence of iron	Fe
gene2573	Scaffold7	BAC0105	*cueR/ybbI*	Regulates the transcription of the copA and cueO genes. It detects cytoplasmic copper stress and activates transcription in response to increasing copper concentrations. CueR is a direct regulator of copZA transcription that mediates copper induction	Cu
gene2574	Scaffold7	BAC0625	*copA*	Copper resistance protein A. Required for the copper-inducible expression of copper resistance; part of the copper resistance determinant system.	Cu
gene2924	Scaffold8	BAC0113	*cutA*	Divalent-cation tolerance protein CutA; Involved in resistance toward heavy metals	Cu
gene2985	Scaffold8	BAC0371	*soxS*	Regulatory protein SoxS; Increases the Zn uptake system znuACB; Transcriptional activator of the superoxide response regulon of *E. coli* that includes at least 10 genes such as sodA, nfo, zwf and micF. Binds the DNA sequence 5'-GCACN7CAA-3'. It also facilitates the subsequent binding of RNA polymerase to the micF and the nfo promoters.	Zn
gene3013	Scaffold8	BAC0470	*zur/yjbK*	Zinc uptake regulation protein zur; Acts as a negative controlling element, employing Zn2+ as a cofactor to bind the operator of the repressed genes (znuACB). Zinc uptake regulation protein.	Zn
gene3288	Scaffold10	BAC0049	*bhsA/ycfR/comC*	Seems to be involved in the regulation of biofilm formation. May decrease biofilm formation by repressing cell-cell interaction and cell surface interaction	Cu

Gene ID	Location	BacMet ID	Gene	Description	Resistance to Heavy Metal
gene3700	Scaffold12	BAC0334	*robA*	Right origin-binding protein RobA; Organic solvent tolerance by robA is dependent on soxRS; Rob acts as a transcriptional activator. It can confer resistance to antibiotics such as tetracycline, chloramphenicol, phosphomycin, kanamycin, novomycin etc.	Ag, Hg, Cd
gene3751	Scaffold13	BAC0644	*corD*	mutations in apaG/corD give a phenotype of low-level Co2+ resistance. They also decrease Mg2+ efflux but not influx via the CorA Mg2+ transport system.	Co, Mg
gene3846	Scaffold14	BAC0251	*mntH/yfeP*	Divalent metal cation transporter MntH; H+-stimulated, divalent metal cation uptake system. Involved in manganese and iron uptake. Can also transport cadmium, cobalt, zinc and to a lesser extent nickel and copper. Involved in response to reactive oxygen.	Mn, Fe, Cd, Co, Zn
gene3990	Scaffold15	BAC0087	*mgtA*	Magnesium-transporting ATPase, P-type 1; Mediates magnesium influx to the cytosol.	Co, Mg
gene4069	Scaffold16	BAC0307	*pcoE*	Copper-binding protein PcoE; lies distal, downstram from the regulatory genes (gene order in the operon- pcoABCDRSE); Required for the copper-inducible Expression of copper resistance. Activated by the two-component regulatory system CusS/CusR; part of the copper resistance determinant system (pcoABCDRSE)	Cu, Ag
gene4070	Scaffold16	BAC0309	*pcoS*	Sensor protein PcoS; member of a two-component regulatory system PcoS/PcoR. Activate PcoR by phosphorylation; part of the copper resistance determinant system (pcoABCDRSE)	Cu
gene4071	Scaffold16	BAC0308	*pcoR*	Transcriptional regulatory protein PcoR; member of a two-component regulatory system pcoS/pcoR. Involved in the activation of copper resistance gene operon pcoABCD by binding to a specific site on the cop operon promoter (copper box). part of the copper resistance determinant system (pcoABCDRSE)	Cu
gene4072	Scaffold16	BAC0306	*pcoD*	Copper resistance protein D; Required for the copper-inducible expression of copper resistance; part of the copper resistance determinant system (pcoABCDRSE)	Cu
gene4073	Scaffold16	BAC0305	*pcoC*	Copper resistance protein PcoC; required for the copper-inducible expression of copper resistance; located in periplasm; part of the copper resistance determinant system (pcoABCDRSE)	Cu
gene4074	Scaffold16	BAC0304	*pcoB*	Copper resistance protein PcoB; required for the copper-inducible expression of copper resistance; located in cell outer membrane; it's peripheral membrane protein	Cu
gene4075	Scaffold16	BAC0303	*pcoA*	Copper resistance protein PcoA; required for the copper-inducible expression of copper resistance. May have oxidase activity; part of the copper resistance determinant system (pcoABCDRSE)	Cu
gene4079	Scaffold16	BAC0346	*silP*	Silver exporting P-type ATPase silP; Component of the sil cation-efflux system that confers resistance to silver; silP can participate in resistance to Ag+ individually; located in cell membrane.	Ag
gene4081	Scaffold16	BAC0107	*cusA/ybdE*	Part of a cation efflux system (CusA, CusB, CusC and CusF) that mediates resistance to copper and silver; located in cell inner membrane; Belongs to the AcrB/AcrD/AcrF family	Cu, Ag
gene4082	Scaffold16	BAC0342	*silB*	SilB, a membrane fusion protein that brings together the inner and outer membranes of Gram-negative bacteria; Component of the sil cation-efflux system (silABC) that confers resistance to silver	Ag

Gene ID	Location	BacMet ID	Gene	Description	Resistance to Heavy Metal
gene4083	Scaffold16	BAC0345	*silF*	Silver efflux system periplasmic chaperone; It is a small gene resides in between SilC and SilB of SilCFBA(ORF105)P operon; It carries Ag+ from its periplasmic site of release by SilP to the periplasmic uptake site of SilA, as part of the SilCBA complex	Ag
gene4084	Scaffold16	BAC0343	*silC*	SilC is an outer membrane lipoprotein; Component of the sil cation-efflux system (silABC) that confers resistance to silver	Ag
gene4085	Scaffold16	BAC0111	*cusR/ylcA*	Member of the two-component regulatory system CusS/CusR. Activates the Expression of cusCFBA and plasmid pRJ1004 gene pcoE in response to increasing levels of copper ions, and probably silver ions. Can also increase the basal-level Expression of copper resistance gene operon pcoABCD; located in cytoplasm	Cu, Ag
gene4086	Scaffold16	BAC0348	*silS*	silS encodes a membrane sensor kinase; Component of the sil cation-efflux system that confers resistance to silver. Probable member of a two-component regulatory system SilS/SilR. May activate SilR by phosphorylation	Ag
gene4087	Scaffold16	BAC0344	*silE*	Component of the sil cation-efflux system that confers resistance to silver; it also can participate in resistance to Ag+ individually	Ag
gene4166	Scaffold17	BAC0318	*pstS*	A periplasmic phosphate binding protein; Part of the ABC transporter complex PstSACB involved in phosphate import.	As
gene4167	Scaffold17	BAC0317	*pstC*	An integral membrane permease protein; Part of the binding-protein-dependent transport system for phosphate; probably responsible for the translocation of the substrate across the membrane.	As
gene4168	Scaffold17	BAC0315	*pstA*	An integral membrane protein. Part of the binding-protein-dependent transport system for phosphate; probably responsible for the translocation of the substrate across the membrane.	As
gene4169	Scaffold17	BAC0316	*pstB*	Phosphate transporting ATPase; Part of the ABC transporter complex PstSACB involved in phosphate import. Responsible for energy coupling to the transport system.	As
gene4176	Scaffold17	BAC0541	*yieF*	A chromate reductase. Reduces chromate (Cr (VI)) to Cr (III)	Cr, V, Mo
gene4238	Scaffold18	BAC0136	*dsbA*	Disulfide oxidoreductase; Required for disulfide bond formation in some periplasmic proteins such as PhoA or OmpA. Acts by transferring its disulfide bond to other proteins and is reduced in the process. DsbA is reoxidized by DsbB. It is required for pilus biogenesis	Cd, Zn, Hg
gene4288	Scaffold18	BAC0368	*sodA*	Mn-dependent superoxide dismutase; Destroys superoxide anion radicals which are normally produced within the cells and which are toxic to biological systems.	Se
gene4544	Scaffold21	BAC0086	*corA*	Mediates influx of magnesium ions. Can also mediate cobalt and manganese uptake, albeit only at extracellular concentrations that are toxic to the cell. Does not transport iron.	Mg, Co, Ni, Mn
gene4833	Scaffold27	BAC0589	*arsR*	Transcriptional repressor for the ars operon. ArsR is a trans-acting regulatory protein which controls its own expression. The repressive effect of ArsR is alleviated by oxyions of +III oxidation state of arsenic, antimony, and bismuth, as well as arsenate (As(V)).	As, Sb, Bi
gene4834	Scaffold27	BAC0033	*arsD*	An arsenic metallochaperone for an arsenic detoxification pump	As

Gene ID	Location	BacMet ID	Gene	Description	Resistance to Heavy Metal
gene4835	Scaffold27	BAC0030	*arsA*	Anion-transporting ATPase; Determinants of a membrane-associated oxyanion-stimulated ATPase protein; energizes the arsenite efflux pump by ATP hydrolysis; Catalyzes the extrusion of the oxyanions arsenite, antimonite and arsenate. Maintenance of a low intracellular concentration of oxyanion produces resistance to the toxic agents.	As, Sb
gene4836	Scaffold27	BAC0031	*arsB*	Arsenic pump membrane protein, located in cell inner membrane. Involved in arsenite and antimonite resistance. Thought to form a channel of an arsenite pump.	As, Sb
gene4837	Scaffold27	BAC0582	*arsC*	Reduction of arsenate [As(V)] to arsenite [As (III)]. This protein expands the substrate specificity of ArsAB pump which can extrude arsenite and antimonite to allow for arsenate pumping and resistance.	As, Sb
gene4852	Scaffold27	BAC0163	*fecD*	Fe(3+) dicitrate transport system permease protein FecD; Part of the binding-protein-dependent transport system for citrate-dependent Fe3+. Probably responsible for the translocation of the substrate across the membrane.	Ni, Co
gene4864	Scaffold28	BAC0467	*zraR/hydH*	Transcriptional regulatory protein ZraR; Member of the two-component regulatory system ZraS/ZraR. When activated by ZraS it acts in conjunction with sigma-54 to regulate the Expression of zraP. Positively autoregulates the Expression of the zraSR operon.	Zn
gene4904	Scaffold29	BAC0643	*corB*	Magnesium-transporting ATPase, P-type 1; Mediates magnesium influx to the cytosol.	Co, Mg
gene4980	Scaffold31	BAC0462	*zntR/yhdM*	Zinc-responsive transcriptional regulator zntR; regulates the zntA	Zn
gene5020	Scaffold33	BAC0111	*cusR/ylcA*	Member of the two-component regulatory system CusS/CusR. Activates the Expression of cusCFBA and plasmid pRJ1004 gene pcoE in response to increasing levels of copper ions, and probably silver ions. Can also increase the basal-level Expression of copper resistance gene operon pcoABCD; located in cytoplasm	Cu, Ag
gene5024	Scaffold33	BAC0107	*cusA/ybdE*	Part of a cation efflux system (CusA, CusB, CusC and CusF) that mediates resistance to copper and silver; located in cell inner membrane; Belongs to the AcrB/AcrD/AcrF family	Cu, Ag

Note: BacMet, the metal resistance genes database (http://bacmet.biomedicine.gu.se/index.html).
